# Engineering human bone marrow-derived mesenchymal stromal cell aggregates for enhanced extracellular vesicle secretion in a vertical-wheel bioreactor

**DOI:** 10.3389/fbioe.2025.1664302

**Published:** 2026-01-16

**Authors:** Danyale Berry, Breana Boirie, Mandip Singh, Li Sun, Sunghoon Jung, Yan Li, Changchun Zeng

**Affiliations:** 1 Department of Industrial and Manufacturing Engineering, FAMU-FSU College of Engineering, Florida Agricultural and Mechanical University, Tallahassee, FL, United States; 2 FAMU-FSU College of Engineering, High Performance Materials Institute, Florida State University, Tallahassee, FL, United States; 3 College of Pharmacy and Pharmaceutical Sciences, Florida Agricultural and Mechanical University, Tallahassee, FL, United States; 4 Department of Chemical and Biomedical Engineering, FAMU-FSU College of Engineering, Florida State University, Tallahassee, FL, United States; 5 Department of Biomedical Sciences, College of Medicine, Florida State University, Tallahassee, FL, United States; 6 PBS Biotech Inc., Camarillo, CA, United States

**Keywords:** 3D aggregates, EV biogenesis, extracellular vesicles, human mesenchymal stem cells, neural inflammation, vertical-wheel bioreactor

## Abstract

**Introduction:**

Human mesenchymal stem/stromal cells (hMSCs) hold significant regenerative potential due to their anti-inflammatory and pro-angiogenic secretome. Three-dimensional (3D) hMSC aggregates secrete extracellular vesicles (EVs) with enhanced immunomodulatory properties compared to 2D cultures. However, the clinical translation of hMSC-EVs remains limited by low production yield. This study investigates scalable EV generation from 3D hMSC aggregates in a novel Vertical-Wheel Bioreactor (VWBR), leveraging shear stress-mediated biochemical cues to enhance EV biogenesis and cargo relevant to nerve regeneration.

**Methods:**

Bone marrow-derived hMSCs were cultured as 3D aggregates in VWBRs and exposed to two different culture media—αMEM/FBS (serum-containing) and DMEM/F12/B27 (serum-free)—under three agitation speeds (25, 40, and 64 rpm). Metabolite analysis and qRT-PCR were performed to assess metabolic activity and EV biogenesis, focusing on ESCRT machinery markers. EVs were isolated and evaluated for yield, size, markers, and microRNA cargo. Functional assays were conducted to measure the effects on EVs on Schwann cells under LPS-induced neural inflammation.

**Results:**

VWBR culture resulted in increased expression of EV biogenesis genes and glycolytic pathway markers compared to static culture. The αMEM/FBS (serum-containing) condition was more robust than DMEM/F12/B27 (serum-free) condition. EV yield (EV number per cell) increased by 3-10 fold (in serum-containing medium) in VWBR compared to static culture, with particle sizes ranging from 120-180 nm and appropriate EV marker expression. microRNA-sequencing showed upregulation of miR-29a-3p, miR-451a, miR-224-5p, miR-16-5p, miR-133a-3p, and miR-143-3p, indicating enhanced EV biogenesis, metabolic reprogramming, and immunomodulatory potential. Functionally, VWBR-derived EVs modulated inflammatory gene expression in Schwann cells exposed to LPS.

**Discussion:**

VWBR-driven hydrodynamics promotes EV biogenesis from 3D hMSC aggregates, improving metabolic activity, EV cargo relevance, and functional efficacy. The resulting EVs exhibit therapeutic cargo capable of modulating neural inflammation. These findings advance understanding of dynamic aggregation on metabolic cues and EV production, demonstrating a scalable strategy for generating therapeutically potent hMSC-EVs for neuropathic and regenerative applications.

## 1 Introduction

Human mesenchymal stem/stromal cells (hMSCs) hold great potential for treatment in regenerative medicine due to the anti-inflammatory and pro-angiogenic secretome (Yin et al., 2019). Due to safety and efficacy concerns associated with stem cell therapy, cell-free approaches utilizing hMSC-derived extracellular vesicles (EVs) have emerged as promising alternative therapeutics. EVs are spherical, lipid-bilayer particles secreted by nearly all cell types (Kumar et al., 2024). EVs are now recognized for their crucial roles in cell-to-cell and cell-to-matrix communication, tissue homeostasis, and various pathophysiological processes (Berumen Sanchez et al., 2021). These vesicles, particularly small size EV subpopulations ranging from ∼30 to 200 nm in average diameter, contain therapeutic cargo reflective of their cell of origin, including growth factors, lipids, proteins, and nucleic acids (Muok et al., 2024; Ene et al., 2025; van Niel et al., 2022; Math et al., 2019; Liu et al., 2024). The targeted delivery of these bioactive molecules facilitates disease modulation, offering an alternative to traditional, more limited treatment methods (Mehrabadi, 2024).

hMSC-derived EVs have shown therapeutic efficacy both *in vitro* and *in vivo* for treating conditions such as inflammatory autoimmune diseases and neurodegenerative disorders (Rather et al., 2023). Li et al. demonstrated that adipose-derived EVs (ADEV) promote the proliferation and migration of dermal papilla cells in alopecia, a dermatological inflammatory disorder (Li et al., 2022). *In vivo*, mice treated with ADEV showed enhanced hair follicle growth and thicker dermis, supported by the activation of Wnt signaling pathway and reduced expression of miR-22 and TNF-α signaling pathways (Li et al., 2022). Zhang et al. utilized human umbilical cord blood MSC (hUCMSC)-derived EVs to promote regenerative wound healing and inhibit scar formation in mice, highlighting the role of miR-21-5p and miR-125-5p in EV cargo (Zhang et al., 2021a). In another study, hUCMSC-derived EVs were combined with a nerve conduit to enhance neuronal neurite growth in rats with sciatic nerve injury, resulting in improved angiogenesis, thicker myelin sheaths, and larger axon diameters compared to the control group (Tang et al., 2024). These studies demonstrate that EVs from hMSCs effectively promote tissue regeneration in various disease models in both *in vitro* and *in vivo* pre-clinical trials. However, the low yield of EV production remains a limitation for clinical applications (Gowen et al., 2020; Grangier et al., 2021).

Traditional two-dimensional (2D) cultures produce a low yield of EVs and batch-to-batch variability between cellular passages (Gowen et al., 2020). Furthermore, 2D culture fails to replicate the native extracellular matrix (ECM) environment, limiting cell-to-cell and cell-to-matrix interactions (Phan et al., 2018). In contrast, three-dimensional (3D) cultures enable cells to form aggregates that more accurately mimic *in vivo* conditions, thereby enhancing EV production (Yuan et al., 2022). Recent studies have highlighted the advantages of culturing hMSCs as 3D aggregates—densely packed spheroids of approximately 500 to 10,000 cells with a hypoxic core that better mimics the *in vivo* microenvironment compared to traditional adherent monolayer cultures (Yuan et al., 2019). In this 3D format, hMSCs exhibit enhanced cell-matrix interactions, signal transduction, paracrine signaling, and therapeutic efficacy (Zhang et al., 2025). These spherical aggregates form tight intercellular junctions facilitated by the expression of adhesion molecules such as N-cadherin, E-cadherin, and integrins, which maximize cell-to-cell communication (Zhang et al., 2025). Moreover, 3D aggregates more closely replicate native ECM microenvironment, thereby improving the functionality and therapeutic potential of hMSC-derived EVs. Helsper et al. demonstrated the therapeutic utility of hMSC aggregates in a rat model of ischemic stroke, showing decreased sodium concentrations following treatment—a potential marker of improved tissue recovery (Helsper et al., 2022). Once formed, the aggregates secrete cytoskeletal and ECM components that stabilize the structure and further enhance paracrine signaling, stemness, differentiation potential, and tissue repair capabilities (Zhang et al., 2025). Given these promising results, hMSC aggregates represent a powerful platform for large-scale EV production, offering notable advantages over 2D cultures, particularly in terms of EV yield and cargo consistency.

Recent studies have utilized Vertical-Wheel Bioreactors (VWBRs) to examine the impact of shear stress on hMSCs cultured on microcarriers, aiming to optimize cell health, enhance metabolite activity, and increase EV biogenesis and cargo expression (Muok et al., 2024; Ene et al., 2025; Jeske et al., 2023; Borys et al., 2021; Lembong et al., 2020; de Sousa Pinto et al., 2019; Otahal et al., 2025). Unlike traditional stirred-tank reactors, the VWBR features a U-shaped vessel that enables even gas and nutrient distribution, ensuring efficient cell suspension while maintaining low shear stress (0.2–0.3 dyn/cm^2^), ∼10 fold lower than traditional stirred tank bioreactors (Borys et al., 2021; Lembong et al., 2020; de Sousa Pinto et al., 2019; Sousa et al., 2015). This design minimizes mechanical forces that could be too harsh for shear-sensitive hMSCs (Borys et al., 2021; Lembong et al., 2020; de Sousa Pinto et al., 2019; Sousa et al., 2015). While previous studies have investigated hMSCs expanded on microcarriers within the VWBR, the behavior and EV production capacity of hMSC 3D aggregates in VWBR remain unexplored.

Dynamic 3D culture conditions, particularly those involving shear stress, have been shown to improve EV yield and enhance the expression of therapeutic cargo (Gowen et al., 2020; Jeske et al., 2023; Yuan et al., 2022). hMSC aggregation has not been successful in traditional spinner flask culture based on our previous experience (Ma et al., 2025). Our previous study compared the effect of shear stress within the VWBR to 2D static culture, investigating the secretion and cargo profile of hMSC-derived EVs (Jeske et al., 2023). hMSC-derived EVs produced under shear stress in the VWBR exhibited elevated levels of EV markers (HRS, syntenin-1, CD81, and CD63) and a significant upregulation of therapeutic miRNAs, including miR-10, 19a, 19b, 21, 30b, 92a, 126, and 132, compared to static 2D culture (Gowen et al., 2020; Jeske et al., 2023; Yuan et al., 2022). The study indicates that shear stress within VWBRs upregulates the endosomal sorting complex required for transport (ESCRT)-dependent and ESCRT-independent EV biogenesis markers compared to 2D culture, leading to increased secretion of miRNAs associated with angiogenesis, wound healing, and neuroprotection (Jeske et al., 2023). These findings underscore the importance of optimizing both biochemical and mechanical culture conditions to enhance EV production and therapeutic efficacy.

This study aims to advance the understanding of dynamic aggregation and metabolic influences on EV production, improving preconditioning techniques for bone marrow hMSC secretome enhancement. This study investigates the influence of biochemical cues and shear stress on EV secretion from 3D hMSC aggregates and cargo expression using the VWBR, focusing on nerve regeneration and neuropathic treatment (Muok et al., 2024; Jeske et al., 2023). The hypothesis of this study is that the low shear environment of VWBR enables the bone marrow hMSC aggregation for EV generation, while media composition would modulate EV cargo profiles and metabolic state. In this study, two types of culture media were utilized to examine the impact of biochemical cues on EV production: αMEM/FBS, a standard MSC serum-containing growth medium, and serum-free DMEM/F12/B27, commonly utilized in cellular differentiation and containing additional amino acids, vitamins, and growth factors that may influence EV cargo composition and metabolic state. Shear stress was applied through three agitation speeds (25, 40, and 64 rpm), selected based on our previous work demonstrating that these parameters promote increased EV biogenesis while maintaining cell viability (Jeske et al., 2023). hMSCs were characterized through metabolite analyses and gene expression, focusing on EV ESCRT machinery markers. microRNA-sequencing for 3D hMSC EV cargo was analyzed. *In vitro* inflammation studies were conducted using the Schwann cell-based functional assay. Together, these investigations were expected to provide a systematic framework for understanding how dynamic culture and microenvironmental factors influence bone marrow hMSC EV biogenesis, therapeutic cargo, and functional efficacy. This study established a scalable culture system to produce EVs from hMSC 3D aggregates as cell-free therapeutics for treating neurological disorders.

## 2 Materials and methods

### 2.1 hMSC 2-D culture expansion

**FIGURE 1:**
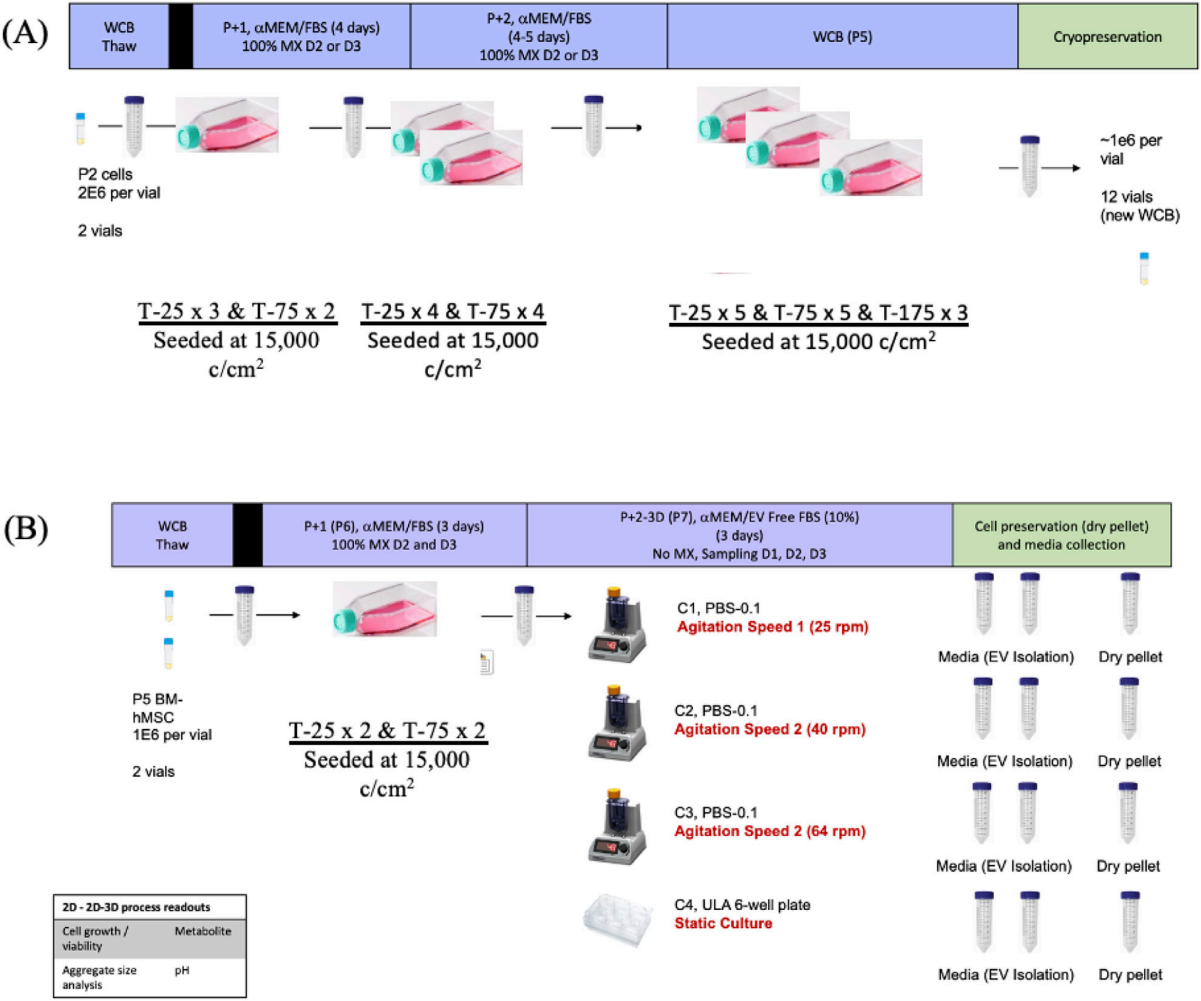
Schematic illustration of the experimental design. Experimental illustrations to establish Working Cell Bank (WCB) for the entire experiment **(A)** and flow of experiment from thaw to harvest from bioreactor culture **(B)**.

Bone marrow-derived hMSCs (BM-hMSCs, purchased from Lonza Walkersville Inc.) isolated from healthy donor human bone marrow mononuclear cells were cryopreserved at passage two in CryoStor CS10 (StemCell Technologies, Vancouver, Canada) for long-term storage (Jeske et al., 2023). To establish a working cell bank (WCB) for the study, BM-hMSCs were thawed and seeded at a density of 15,000 cells/cm^2^ into Corning Cell-BIND T-25 cm^2^, T-75 cm^2^, and T-175 cm^2^ flasks. Cells were maintained under standard conditions (37 °C, 5% CO_2,_ 20% O_2_) in complete culture medium (CCM) of α-Minimum Essential Medium (αMEM, Thermo Fisher Scientific Inc., Waltham, MA, USA) supplemented with 10% fetal bovine serum (FBS, Atlanta Biologicals, Lawrenceville, GA) and 1% Penicillin/Streptomycin. Media changes were performed on days 2 and 3, then harvested and passaged on day 4 utilizing 0.05% Trypsin-EDTA (Thermo Fisher Scientific). At passage 5, all cells were cryopreserved at 4 × 10^6^ cells per vial in CryoStar CS10 for subsequent experiments, as illustrated in Figure 1A.

### 2.2 Aggregate-based hMSC culture expansion in vertical-wheel bioreactors

For each experiment, two vials from the WCB (4 × 10^6^ cells per vial) were thawed and seeded into Corning Cell-BIND T-25 cm^2^ and T-75 cm^2^ within CCM at passage 6. Once confluent, 0.05% Trypsin-EDTA was utilized to harvest BM-hMSCs, and assessed for cell number and viability using the Via 1-Cassette™ (ChemoMetec, Bohemia, NY, USA) to ensure an adequate number of cells for seeding into the VWBR (PBS Biotech Inc., Camarillo, CA, USA). The 0.1 L vessels were first filled to the seeding volume of 80 mL with either EV-depleted αMEM/FBS (Experiment 1) as serum-containing medium or Dulbecco’s Modified Eagle Medium: Nutrient Mixture F12 (DMEM/F12) (Thermo Fisher Scientific) plus 2% B27 serum-free supplement (Thermo Fisher Scientific), referred as DMEM/F12/B27 (Experiment 2) as serum-free medium. The EV-depleted FBS was prepared through ultracentrifugation (i.e., FBS was ultracentrifuged at 100,000 g for 18 h to remove particle and EVs). Each vessel was inoculated at 20,000 cells/mL, relative to a final working volume of 100 mL/vessel. During inoculation, vessels were agitated at 20 rpm, after which the agitation speed was adjusted to the experimental conditions of 25 rpm, 40 rpm, or 64 rpm. Daily sampling assessed cell count, pH, metabolite levels, and imaging. After 3 days of culture, each vessel was harvested for cellular analysis and EV collection.

Simultaneously with the bioreactor culture, BM-hMSCs were seeded at 100,000 cells/well in an ultra-low attachment (ULA) 6-well plate (Corning, Corning Incorporated Inc.). Cells were cultured under standard conditions in either EV-depleted αMEM/FBS (Experiment 1) or DMEM/F12/B27 (Experiment 2). After 3 days, cells were harvested, and the culture media was collected for EV analysis. Each experiment was conducted in triplicate. The complete experimental plan from WCB thaw to cellular and EV collection is illustrated in Figure 1B. To accurately assess the effect of agitation and media type on cellular viability and proliferation, daily sampling of the VWBR was conducted to analyze the cellular environment throughout the experiment. Daily measurements included cell number, pH value of the media, metabolite measurements, and imaging. Upon harvest, cells were pelleted and frozen for reverse transcription and quantitative polymerase chain reaction (qRT-PCR) analysis, while the culture media was collected for EV isolation. Isolated EVs were characterized with NTA and Western blot to confirm EV marker expression.

### 2.3 Cell number, pH values, and metabolite analysis during bioreactor culture

The agitation speed was temporarily adjusted to 20 rpm to perform sampling from the bioreactors. A total of 3 mL of culture was collected daily (the volume was replenished) over the 3-day experiment for cell count, imaging, and metabolite analysis. 1 mL of the sample was immediately analyzed using the BioProfile Flex2 (Nova Biomedical, Waltham, MA, USA) to assess gas exchange and metabolite consumption in the cell culture. Another 1 mL was loaded into a Via1-Cassette^TM^ and analyzed using the NucleoCounter NC-200 (ChemoMetec, Bohemia, NY, USA) to determine cell count and viability. The remaining 1 mL of media was transferred to a 6-well plate and imaged using a microscope to assess cell morphology and aggregation. For the 2D culture, cells were not imaged or counted daily, but were analyzed upon harvest on the final day of culture.

### 2.4 Cell harvesting and sample processing

To harvest, each bioreactor was removed from agitation and allowed to settle. The media from the VWBRs and ULA 6-well plates were collected and centrifuged at 500 rpm for 5 min to separate the cells from the conditioned media. The collected cell pellets were stored at −80 °C for subsequent mRNA isolation and qRT-PCR analysis. The conditioned media was also stored at −80 °C to allow for EV isolation using differential centrifugation, allowing for the assessment of EV size and concentration in response to cellular metabolism and agitation.

### 2.5 EV isolation by differential ultracentrifugation

Differential ultracentrifugation was used to isolate the BM-hMSC-derived EVs from the collected media. Initially, the media was centrifuged at 500 *g* for 5 min at 4 °C to remove cell debris. The supernatant was collected and recentrifuged at 2000 g for 10 min at 4 °C, followed by a final centrifugation step at 10,000 g for 30 min at 4 °C to further eliminate large vesicles and apoptotic bodies. The remaining supernatant was mixed by inversion with a 16% (wt/vol) polyethylene glycol (PEG) solution at a 1:1 volume ratio and incubated overnight at 4 °C to facilitate EV precipitation. Following overnight incubation, the solution was centrifuged at 10,000 g for 70 min at 4 °C, after which the supernatant was carefully removed, and the pellet was allowed to dry by inverting the conical tube. Each pellet was then resuspended in 1 mL of particle-free PBS and subjected to ultracentrifugation at 100,000 g for 2 h at 4 °C. The final supernatant was removed, and the EV pellet was resuspended in 200 μL of PBS, then stored at −80 °C until further characterization.

### 2.6 Nanotracking particle analysis (NTA)

NTA was performed on the isolated EV samples from each experimental condition to assess particle size distribution and concentration. Each measurement was conducted in triplicate using the ZetaView® (TWIN PMX-220, Ammersee, Germany) instrument to ensure accuracy. The software within the ZetaView® system calculated the mean and mode size distribution and the particle concentration per mL of solution, accounting for the dilution factor. For each measurement, 50 μL of EV sample was diluted into 10 mL of particle-free PBS, resulting in a 200x dilution. The measurement chamber was thoroughly cleaned with particle-free PBS between each sample run to prevent cross-contamination.

### 2.7 Western blot analysis

Western blot analysis was performed to confirm the presence of EVs by identifying specific EV-associated proteins. Protein separation was achieved through gel electrophoresis. Protein content within the EVs was quantified using a Bradford assay (Bio-Rad, Hercules, CA, USA). For the assay, EVs were lysed in radio-immunoprecipitation assay (RIPA) buffer, and protein concentration was determined against a stand curve generated from serial dilutions of bovine serum albumin (BSA) ranging from 50 to 1500 μg/mL. Upon addition of Commassie Brillian Blue G-250 dye, protein-dye binding led to a colorimetric shift from brown to blue, which was measured using a microplate reader at an absorbance level of 660 nm. The resulting absorbance values were calculated based on the BSA standard curve, and the EV lysates were normalized to 20 μg/mL.

Following protein quantification, EV samples were denatured at 95 °C for 10 min, loaded into gel wells, and subjected to electrophoresis at 150 V for 2 h. After separation, the proteins were transferred to a nitrocellulose membrane and stained with Ponceau S to confirm transfer efficiency. The membrane was blocked using 5% skim milk (w/v) in Tris-buffered saline with Tween 20 (TBST) for 1 h, then incubated overnight at 4 °C with primary antibodies (Supplementary Table S1). The next day, membranes were washed with TBST, incubated with secondary antibodies for 1 h, and washed again with TBST. Membranes were then sealed in plastic lamination sheets and imaged with the LI-COR Odyssey (LI-COR) imaging system using chemiluminescence mode to visualize protein bands. In addition to positive EV marker proteins, the absence of calnexin, a negative EV marker for cellular expression was confirmed (Supplementary Figure S1).

### 2.8 Reverse transcription and quantitative polymerase chain reaction (qRT-PCR)

After harvesting, cells were frozen as dry pellets at −80 °C to preserve RNA integrity. Cellular mRNA was isolated using the RNeasy Plus Kit (Qiagen, Valencia, CA, USA) following the manufacturer’s protocol to ensure high-purity RNA free of DNA contamination. The purity of the mRNA concentration was assessed using a Nanodrop spectrophotometer (Thermo Fisher Scientific), ensuring A260/A280 ratios fell within the optimal range (1.8-2.2) for high-quality mRNA. Following isolation, qRT-PCR analysis was conducted to assess gene expression levels. Genes of interest include those involved in EV biogenesis, specifically ESCRT-independent and ESCRT-dependent pathways, as well as genes associated with pentose phosphate metabolism and glycolysis, which are crucial for cellular energy balance and EV secretion. Reverse transcription was carried out according to the manufacturer’s instructions using 2 ng of total mRNA, anchored oligo-dT primers (Operon, Huntsville, AL), and Superscript III (Invitrogen, Carlsbad, CA, USA). The software Oligo Explorer 1.2Primers (Genelink, Hawthorne, NY, USA) was used to design the real-time PCR primers specific for target genes (Supplementary Table S2). For normalization of expression levels, β-actin (ACTB) was used as an endogenous control. Using SYBR1 Green PCR Master Mix (Applied Biosystems, Foster City, CA, USA), real-time PCR reactions were performed on an ABI7500 instrument (Applied Biosystems). The amplification reactions were performed as follows: 2 min at 50 °C, 10 min at 95 °C, and 40 cycles of 95 °C for 15 s and 55 °C for 30 s, and 68 °C for 30 s following with a melt curve analysis. Relative mRNA expression levels were quantified using the comparative Ct (ΔΔCt) method. Ct values of target genes were normalized to the housekeeping gene to obtain ΔCt values. Relative fold change in expression was calculated as $2^{-\left(\Delta {\text{Ct}}_{\text{treatmet}}-\Delta {\text{Ct}}_{\text{control}}\right)}$ where ΔC utilized to quantify relative gene expression, comparing gene expression between bioreactor samples and the control.

### 2.9 Transmission electron microscopy (TEM)

TEM was utilized to confirm the isolated EVs’ morphology, size, and integrity following the methodology as demonstrated in our previous publication (Marzano et al., 2025). This technique provides high-resolution imaging to validate the presence of lipid bilayer-enclosed EVs. Briefly, EV isolates were resuspended in 50–100 μL of sterile-filtered PBS to maintain particle stability. Five μL of intact EVs were carefully pipetted for sample preparation onto Parafilm to prevent excess sample loss. A carbon-coated 400 Hex Mesh Copper grid (Electron Microscopy Sciences, EMS) was then placed coating side down onto each EV droplet using fine-tip forceps and incubated for 1 h at room temperature to allow absorption.

Following incubation, grids were washed three times with sterile-filtered PBS to remove any unbound material. To preserve EV structure, samples were fixed in 2% paraformaldehyde (PFA, EM Grade) for 10 min at room temperature. After fixation, grids were transferred onto a 20-μL drop of 2.5% glutaraldehyde (EM Grade) and incubated for an additional 10 min to enhance structural stability. The samples were then negatively stained using 2% uranyl acetate (EMS grade) for 10 min, which provides contrast for TEM imaging. Grids were incubated in 0.13% methyl cellulose combined with 0.4% uranyl acetate for 10 min before excess solution was carefully removed to further stabilize and embed the EVs. The coated side of the grids was left to air dry before imaging using the Hitachi HT7800 electron microscope housed at Florida State University (Lässer et al., 1940). High-resolution images were captured to analyze EV morphology, ensuring the vesicles exhibited a spherical structure with a distinct lipid bilayer.

### 2.10 Small RNA sequencing for EV miRNA cargo analysis

EV-associated miRs were isolated and sequenced in triplicate. EV samples were treated with RNase (ThermoFisher, AM2294) to final concentration of 50 ng/mL, at room temperature for 30 min. RNase inhibitor (NEB, M0314) and PCR grade water were added to EV samples to make a total volume of 200 μL. miRs were isolated by adding 600 μL Trizol LS (ThermoFisher, 10,296,010) according to manufacturer’s instruction. To increase the yield of small RNAs, three volumes of 100% ethanol and linear acrylamide (VWR, 97063–560) were used instead of isopropyl alcohol and incubation time was also increased to overnight at −20 °C. The isolated RNAs were quantified by Qubit microRNA assay kit (ThermoFisher, Q32880). Small RNA libraries (using equal RNA mass) were generated with NEBNext Multiplex Small RNA Library Prep Set for Illumina (NEB; E7300). To increase yield and prevent primer/adaptor dimer, 3’ SR primer was diluted to 1:5 and ligation time was increased to overnight at 16 °C. Similar to mRNA-seq library preparation, HS DNA chip and KAPA library quantification kit were used before submitting to sequencing by Illumina NovaSeq 6,000 in Florida State University College of Medicine Translational lab.

Raw data for miR-seq were submitted to OASIS online miR analysis tool to identify small RNAs on Human reference genome hg38. Differential expressed miRs were analyzed by both OASIS and miRNet using default settings. RNA-seq data was analyzed by NetworkAnalyst 3.0. Genes with counts less than 10, variance less than 10% and unannotated were filtered and normalized by Log2-counts per million. Differentially expressed genes (DEGs) were identified by DEseq2. Heatmap of globe differential expressed genes and gene enriched pathways were also visualized by the same online tool.

### 2.11 EV-treated schwann cell functional assay

Human Schwann cells (SC), as described in our previous study (Khan et al., 2025), were utilized in this functional assay to evaluate the treatment capabilities of the hMSC-derived EVs isolated from each experimental condition at 25 rpm, 40 rpm, and 64 rpm within the αMEM and DMEM experimental groups. The SCs were seeded at a density of 50,000 cells per well in a 12-well plate, and cultured under standard conditions of 5% CO_2_ and 20% O_2_ at 37 °C. The media consisted of high-glucose DMEM, supplemented with 10% FBS and 1% penicillin-streptomycin. The SCs were treated with lipopolysaccharide (LPS) at a 500 ng/mL concentration for 8 h. Following the LPS treatment, the SCs received hMSC-derived EVs isolated from each experimental condition, at a ratio of 1000:1 EVs per cell, for 24 h (Liu et al., 2024). The dose of EVs was standardized across all conditions to ensure consistent treatment across the experimental groups. After the 24-h EV treatment period, the SCs were harvested for qRT-PCR analysis to assess the expression of key pro-inflammatory and anti-inflammatory genes, investigating EV modulation on SC inflammatory response.

### 2.12 miR-21 loading to hMSC EVs

hMSC EVs (12 μL, 1 × 10^10^ particles/µL) were mixed with miR-21 oligo (8 μL, 100 µM) diluted in 200 µL Electroporation buffer (HEPES pH 7.4, 10 mM, Sucrose 285 mM, MgCl_2_ 1.5 mM). The mixture was transferred into the cuvettes (0.4 cm), and electroporated on the Gene Pulser Xcell system (BD) with the setting of square wave, 750V, 5 m length, 10 pulses, 5 s interval. After electroporation, the mixture was incubated with RNase A (0.5 mg/mL) at 37 °C for 20 min to remove un-incorporated miR oligos. hMSC EVs without miR-21 oligo, and only miR-21 oligo was performed at the same time and procedure as control. Total RNA was isolated by Trizol, quantified by Qubit small RNA quantification kit and miR-21 level was quantified by qScript miR quantification kit with miR-21 specific primer.

### 2.13 Statistical analysis

Each bioreactor run was performed independently in triplicate to ensure reproducibility and reliability of the results. The data are presented as means ± standard deviation (SD). For RT-PCR analysis, raw Ct values were calculated into relative expression values using ΔΔCt method and expressed as log_2_ fold changes, which improves normality and stabilizes variance across groups. A one-way analysis of variance (ANOVA) was conducted to assess statistical differences between groups, followed by Tukey’s *post hoc* test for pairwise comparisons of multiple groups. A Student’s t-test was applied to determine statistical significance for direct comparisons between two conditions. A p-value <0.05 was considered statistically significant.

## 3 Results

### 3.1 BM-hMSC aggregation within VWBRs

**FIGURE 2:**
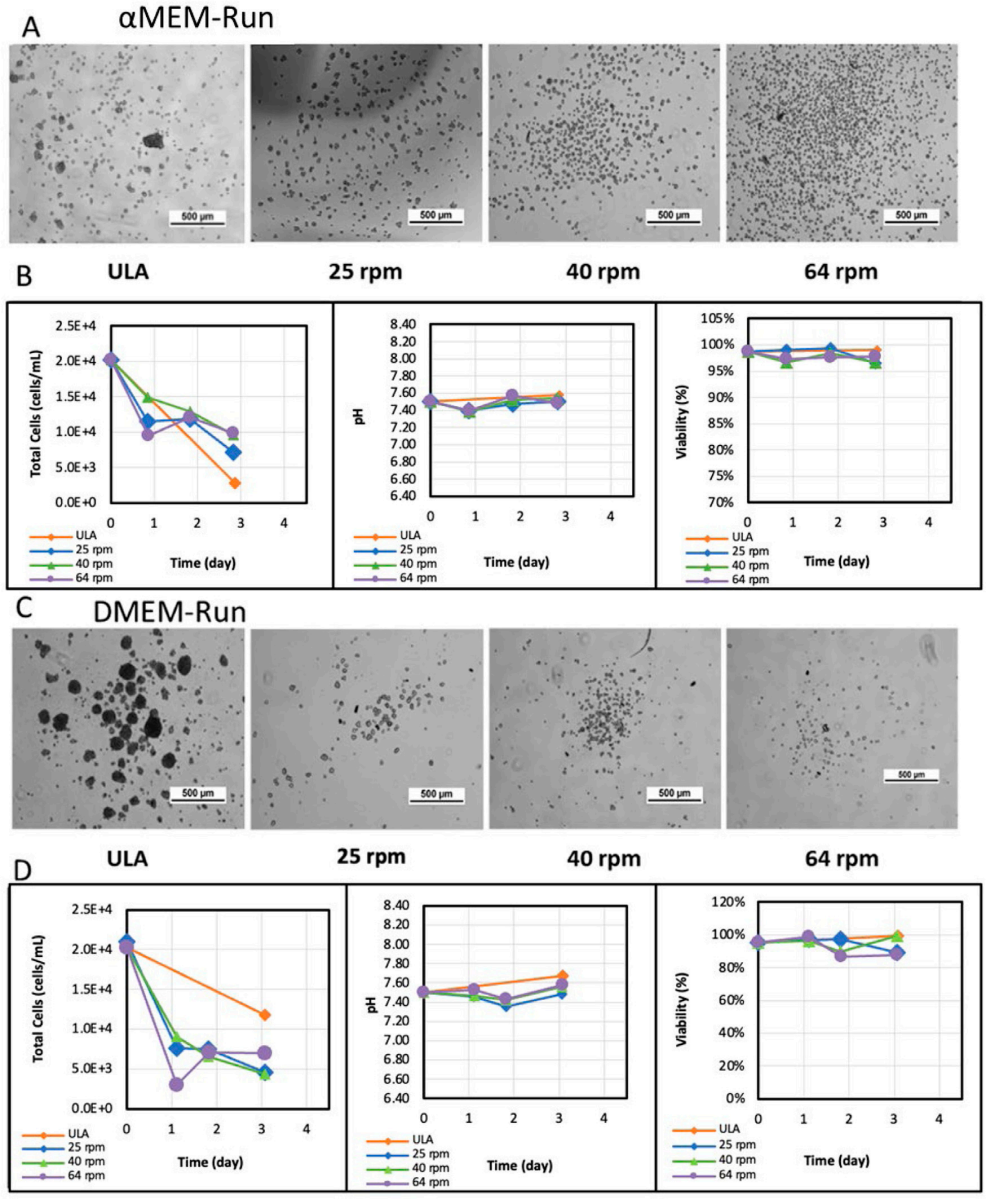
BM-hMSC aggregate images, total cell count, pH, and cellular viability for VWBR runs. Experiment condition one within αMEM/FBS media: **(A)** cell morphology, and **(B)** cellular behaviors: cell concentration, pH value of the spent culture media, and cell viability. Experiment condition 2 with DMEM/B27 media: **(C)** cell morphology, and **(D)** cellular behaviors: cell concentration, pH value of the spent culture media, and cell viability. Note: Daily counts for ULA were not performed due to sampling constraints; endpoint only.

BM-hMSCs were expanded in Corning CellBIND T-flasks to establish a working cell bank (WCB, Supplementary Figure S2). A vial from the WCB was thawed, expanded, and inoculated into PBS 0.1 L bioreactor systems according to the manufacturer’s protocol, with ULA 6-well plate cultures as static controls. To evaluate the impact of different culture conditions on EV production, BM-hMSC aggregates were subjected to agitation speeds of 25, 40, and 64 rpm within αMEM/FBS (serum-containing) or DMEM/F12/B27 (serum-free) based media. After 3 days, cells and EVs from each vessel were harvested. Each experiment was conducted independently in triplicate. Data from the αMEM-Run 1 and DMEM-Run 3 experimental groups are presented as representative results with additional runs in the Supplementary Material (Figure 2; Supplementary Figures S3, S4).

hMSC aggregates were formed for all the conditions of αMEM/FBS conditions (Figure 2A; Supplementary Figure S3). VWBR conditions had more uniform size of hMSC aggregates than the ULA static control. At higher agitation speeds, hBM-MSCs exhibited reduced formation of large aggregates, suggesting that increased turbulence influences aggregate morphology and size. Daily sampling of the bioreactors revealed a progressive decrease in cell count per mL of media across all conditions (Figure 2B). At inoculation, the initial seeding density in all conditions was 2.0 × 10^4^ cells/mL. Cell numbers steadily declined by approximately 0.5-fold per day throughout the experiment, while viability remained consistently high, between 97%–99% for αMEM cultures and 80%–84% for DMEM cultures following initial measurement after inoculation. The pH of the culture environment fluctuated between 7.4 and 7.5, with no significant differences observed between conditions. In DMEM/F12/B27 conditions, VWBR conditions produced uniform and small hMSC aggregates compared to the ULA static control (Figure 2C; Supplementary Figure S4). Cell numbers, pH values, and viability showed the similar trends to the αMEM/FBS conditions (Figure 2D). The ULA 6-well condition did not have sufficient samples for these analyses, but this condition has been reported in our previous study (Jeske et al., 2022).

### 3.2 Analysis of BM-hMSC aggregate metabolism and EV biogenesis

**FIGURE 3:**
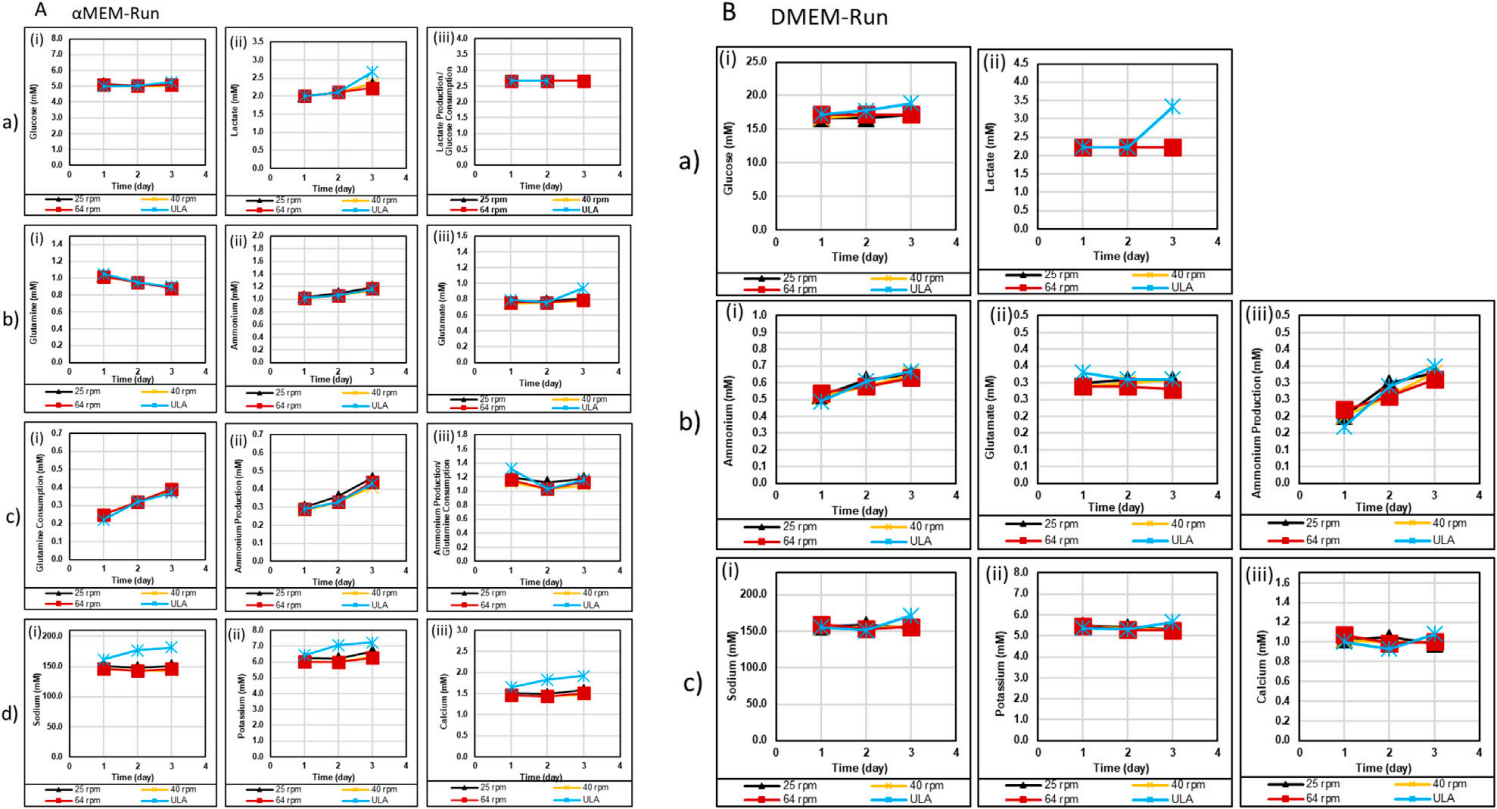
Metabolite analysis acquired from BioProfile Flex2 for αMEM/FBS Run **(A)** and DMEM/F12/B27 Run **(B)**. In **(A) (a)** glucose (i) and lactate (ii) concentrations; (iii) Lactate Production/Glucose Consumption ratio (mol/mol); **(b)** glutamine (i), ammonia (ii), and glutamate (iii) concentrations; **(c)** glutamine consumption (i) and ammonia production (ii) concentrations; (iii) ammonia Production/Glutamine Consumption ratio (mol/mol); **(d)** iron concentrations: (i) Sodium, (ii) Potassium; (iii) Calcium. In **(B) (a)** glucose (i) and lactate (ii) concentrations; **(b)** ammonia (i) and glutamate (ii) concentrations; (iii) ammonia production; **(c)** iron concentrations. (i) Sodium, (ii) Potassium; (iii) Calcium. In **(A) (a)** ULA day 3 lactate production to glucose consumption ratio cannot be determined since the glucose consumption was undetectable (i.e., with a value of ∼0).

Metabolite data revealed distinct metabolic adaptations across culture conditions in VWBR conditions compared to static controls. In αMEM/FBS cultures, lactate levels progressively increased, particularly at 64 rpm (Figure 3Aa). The ULA group showed higher lactate concentration at day 3 than the VWBR condition. Glucose consumption remained stable across all conditions, supporting sustained metabolic activity regardless of agitation speed. The lactate production to glucose consumption mol/mol ratio (the theoretical range is 1.0–2.0) was ∼2.7 which may be influenced by low overall glucose utilization, consistent with enhanced anaerobic metabolism. Collectively, these trends suggest a shift toward glycolytic metabolism under dynamic culture, marked by increased lactate production. For glutamine metabolism, concentrations declined from 1.0 mM to ∼0.8 mM in all conditions (Figure 3Ab). Ammonia levels increased while glutamate rose slightly, yielding an ammonia/glutamine mol/mol ratio of ∼1.0–1.2 (Figure 3Ac). Ion concentrations were notably higher in ULA cultures compared to VWBRs, including sodium (∼170 mM vs. 150 mM), potassium (∼7.0 mM vs. 6.0 mM), and calcium (∼1.8 mM vs. 1.5 mM).

In DMEM/F12/B27 cultures with high baseline glucose concentrations (Figure 3Ba), significant glucose consumption cannot be detected; therefore lactate production/glucose consumption ratios could not be calculated. Lactate production remained consistent, aside from a notable outlier at 40 rpm. Glutamine levels were not able to be measured due to the use of Glutamax. Though ammonia accumulation was observed across all conditions (Figure 3Bb). Ion concentrations were similar between ULA and VWBR groups, with sodium at ∼160 mM, potassium at ∼5.5 mM, and calcium at ∼1.0 mM (Figure 3Bc). Differences in metabolite trends likely reflect the different nutrient composition of DMEM/F12/B27, which contains more vitamins and amino acids than the αMEM formulation, which is supplemented by nutrients of FBS. Based on metabolite data and culture morphology, DMEM/F12/B27 conditions did not support as robust hMSC aggregate formation as αMEM/FBS conditions. The aMEM/FBS conditions were focused in the subsequent experiments.

**FIGURE 4:**
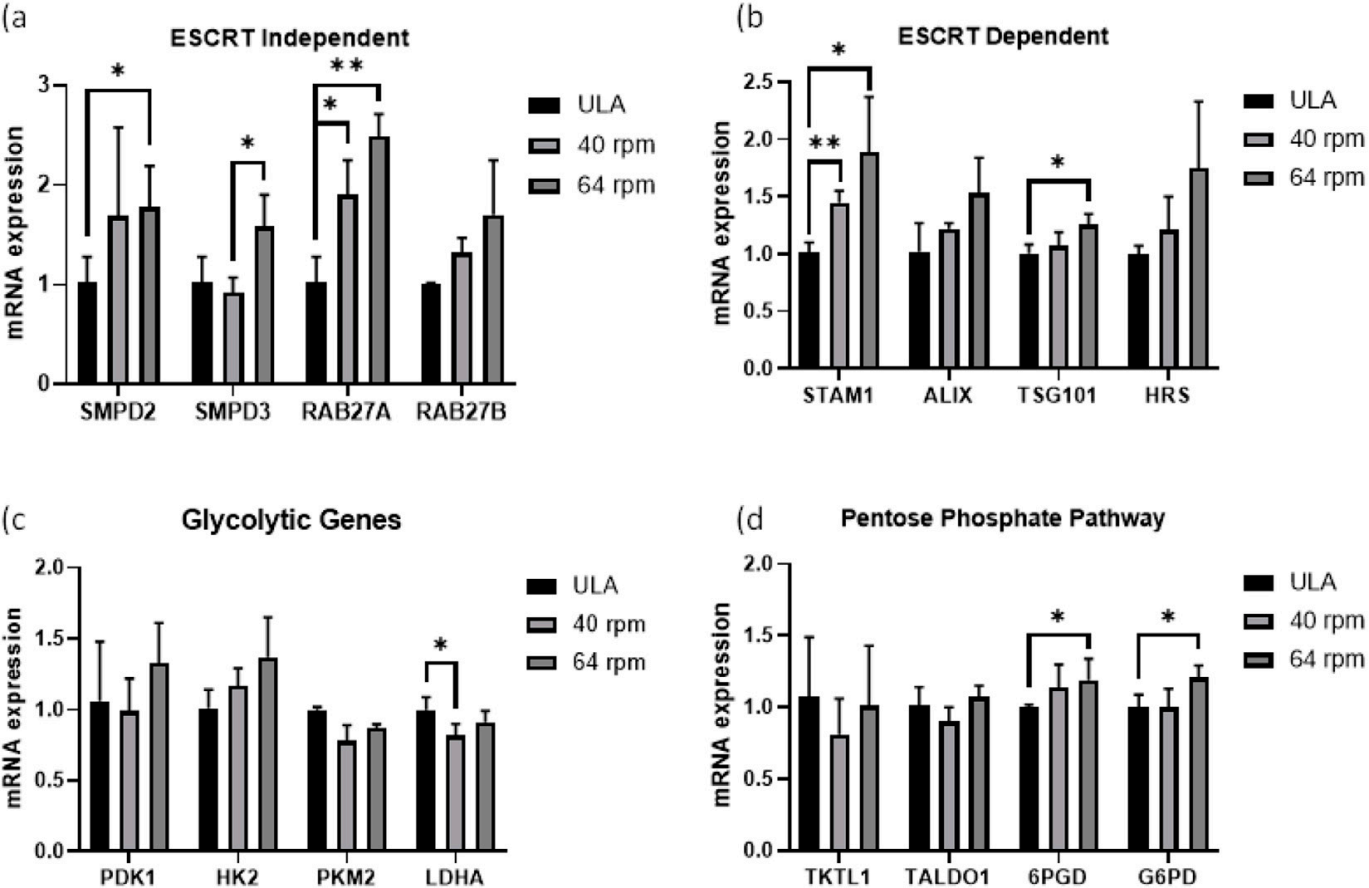
qRT-PCR analysis for mRNA expression of EV biogenesis and metabolic markers in hBM-MSCs. The comparison was performed for VWBR culture (agitation speeds of 40 and 64 rpm) to the static ULA control within αMEM-Run. **(a)** ESCRT independent EV biogenesis genes; **(b)** ESCRT dependent EV biogenesis genes; **(c)** glycolytic genes, **(d)** Pentose phosphate pathway genes. N = 3 (biological replicates from representative bioreactor run), * indicates p < 0.05 and ** indicates p < 0.01.

Following mRNA isolation, qRT-PCR data revealed that BM-hMSCs cultured in αMEM/FBS at 64 rpm exhibited the highest expression of EV biogenesis compared to the static control (ULA 6-well plate) and 40 rpm condition (Figure 4). The 25 rpm did not get sufficient mRNA for the analysis. Among ESCRT-independent genes, *SMPD2*, *SMPD3*, *RAB27a*, and *RAB27b* were all significantly upregulated (∼1.5–2.5-fold) under dynamic VWBR conditions (64 rpm) compared to ULA (Figure 4a). *SMPD3* expression was elevated at 64 rpm relative to 40 rpm, while *RAB27a* and *RAB27b* were significantly upregulated under both dynamic conditions. *SMPD3* enhances EV generation, and *RAB27a* promotes EV docking to the plasma membrane, further supporting the role of dynamic culture in promoting EV release. The analyzed ESCRT-dependent genes include *STAM1, ALIX, TSG101*, and *HRS*, essential for endosomal budding, selective cargo sorting, and MVB formation leading to EV release. *STAM1*, *TSG101,* and *HRS* were significantly upregulated (∼1.3-2 fold) in dynamic VWBR cultures compared to ULA, with *TSG101* showing an additional increase at 64 rpm (Figure 4b). *TSG101* is critical for membrane budding during EV formation, while *STAM1* supports selective cargo sorting. *ALIX* expression did not show statistically significant change compared to the ULA control. Collectively, VWBR promoted 7 out of 8 EV biogenesis genes for αMEM/FBS conditions. For DMEM/F12/B27 conditions, VWBR promoted 4 out of 8 EV biogenesis genes including *Rab27a*, *SMPD2, SMPD3*, and *STAM1*, in particular at 40 rpm (Supplementary Figure S5).

Metabolic gene expression showed modest changes. *PDK1* and hexokinase-2 (*HK2*) were not statistically different across conditions, whereas *LDHA* and *PKM2* were slightly higher in ULA compared to 40 rpm VWBR (Figure 4c). In contrast, *6PGD* and *G6PD*, a key enzyme of the pentose phosphate pathway (PPP), were significantly upregulated at 64 rpm compared to the ULA static condition (Figure 4d). The observed increase in PPP-related gene expression under higher agitation conditions reflects both enhanced glycolytic flux for ATP generation and PPP activation for NADPH production indicating a shift toward redox regulation and metabolic adaptation. In particular, upregulation of *G6PD* in αMEM/FBS cultures supports increased NADPH production via PPP activation (Zhang et al., 2021b). Taken together, these results show that αMEM/FBS cultures in VWBR favor glycolysis and PPP activation. DMEM/F12/B27 runs were not analyzed due to insufficient samples.

### 3.3 Analysis of secreted EVs for size, yield, morphology, and EV markers

**FIGURE 5:**
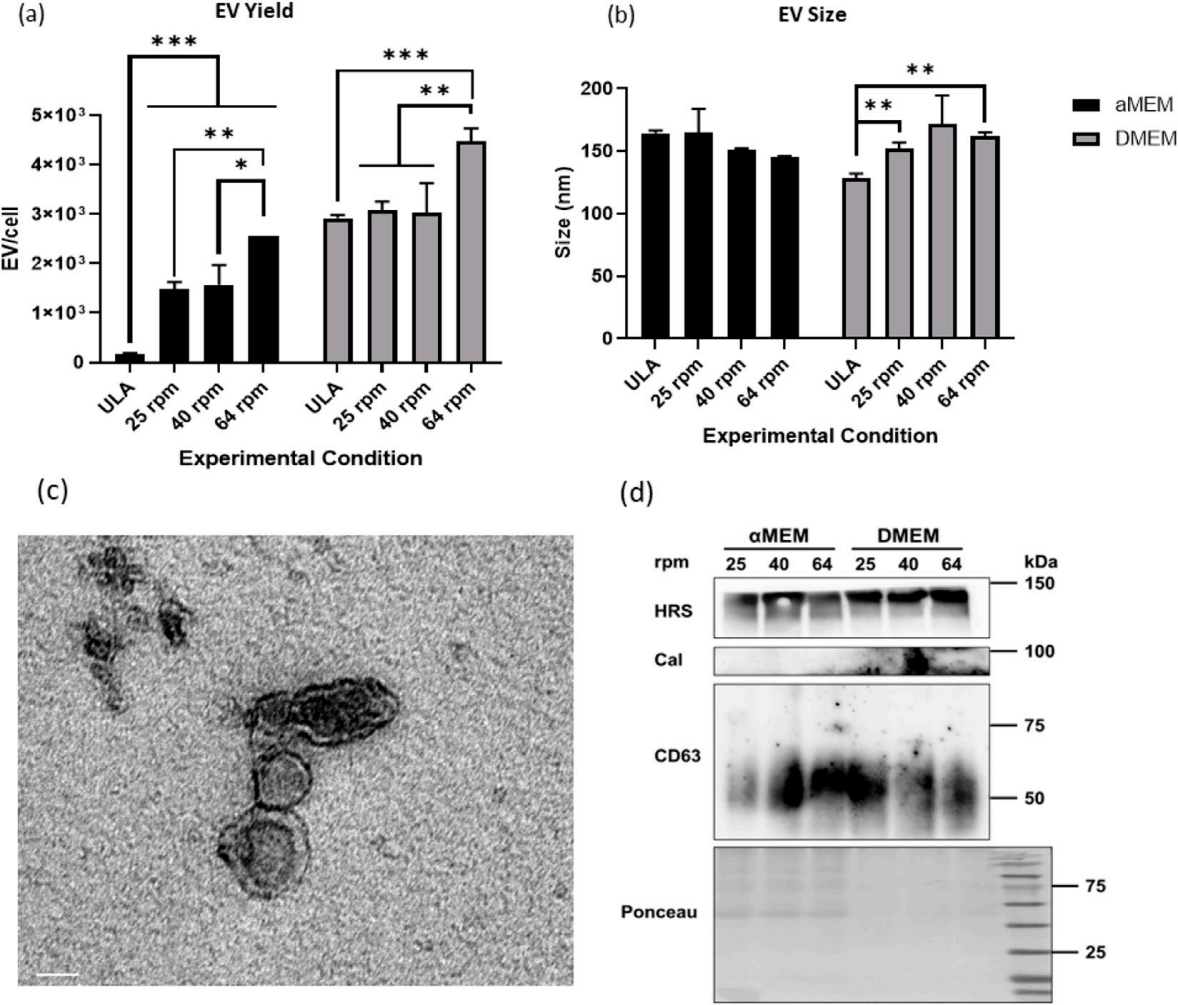
Analysis of secreted EVs for size, yield, morphology and EV markers. NTA data from EV isolation from each experimental condition, including size **(a)** and concentration **(b)**. **(c)** TEM image for EV morphology. Scale bar: 60 nm. **(d)** Western blot analysis for EV markers. N = 3 (biological replicates from three bioreactor runs), * indicates p < 0.05 and ** indicates p < 0.01. ***indicates p < 0.001.

The EVs were isolated from the spent media and characterized by NTA for EV size and yield (Figure 5; Supplementary Figure S6; Supplementary Table S3). EV yields in αMEM/FBS increased significantly with higher agitation speeds, from 2 × 10^2^ EVs per cell at ULA control to 1.5-2.5 × 10^3^ EVs per cell at 64 rpm, representing a ∼10-fold increase (Figure 5a). These findings indicate that dynamic culture promotes EV release. In contrast, within DMEM/F12/B27, EV secretion showed a modest increase with agitation, with the 64 rpm condition reaching significance (from 3 × 10^3^ to 4.5 × 10^3^ EVs per cell) compared to the ULA control. Additionally, average EV size was consistent across all culture conditions in the range of 30–200 nm. αMEM/FBS conditions had EV size of 140–160 nm. For DMEM/F12/B27 conditions, EV size slightly increased under VWBR conditions (140 nm–150–160 nm) (Figure 5b). These results indicate that mechanical stimulation in VWBRs does not alter EV size or membrane integrity, but increases the EV yield, consistent with previous finding from our group (Jeske et al., 2023).

TEM imaging verified the intact concave shape morphology of EVs, reinforcing the reliability of the isolation process of differential centrifugation (Figure 5c). Western blot analysis confirms the presence of positive EV markers, *CD63* and *HRS*. In contrast, the absence of *Calnexin* (negative EV marker) signal confirmed low cellular contamination (Figure 5d). The combination of media composition and mechanical agitation reflects its influence on cellular metabolism and ultimately EV secretion. While PPP-related gene expression in αMEM/FBS was higher under dynamic 3D culture conditions, direct comparison to DMEM/F12/B27 could not be performed due to limitations in RNA yield from these samples as hMSC aggregation in serum-free medium is not robust. For DMEM/F12/B27 conditions, an inverse relationship is observed between EV concentration and size with increasing agitation speed, highlighting the impact of mechanical shear force on vesicle secretion in a serum-free medium.

### 3.4 Analysis of miRNA cargo in the secreted EVs

**FIGURE 6:**
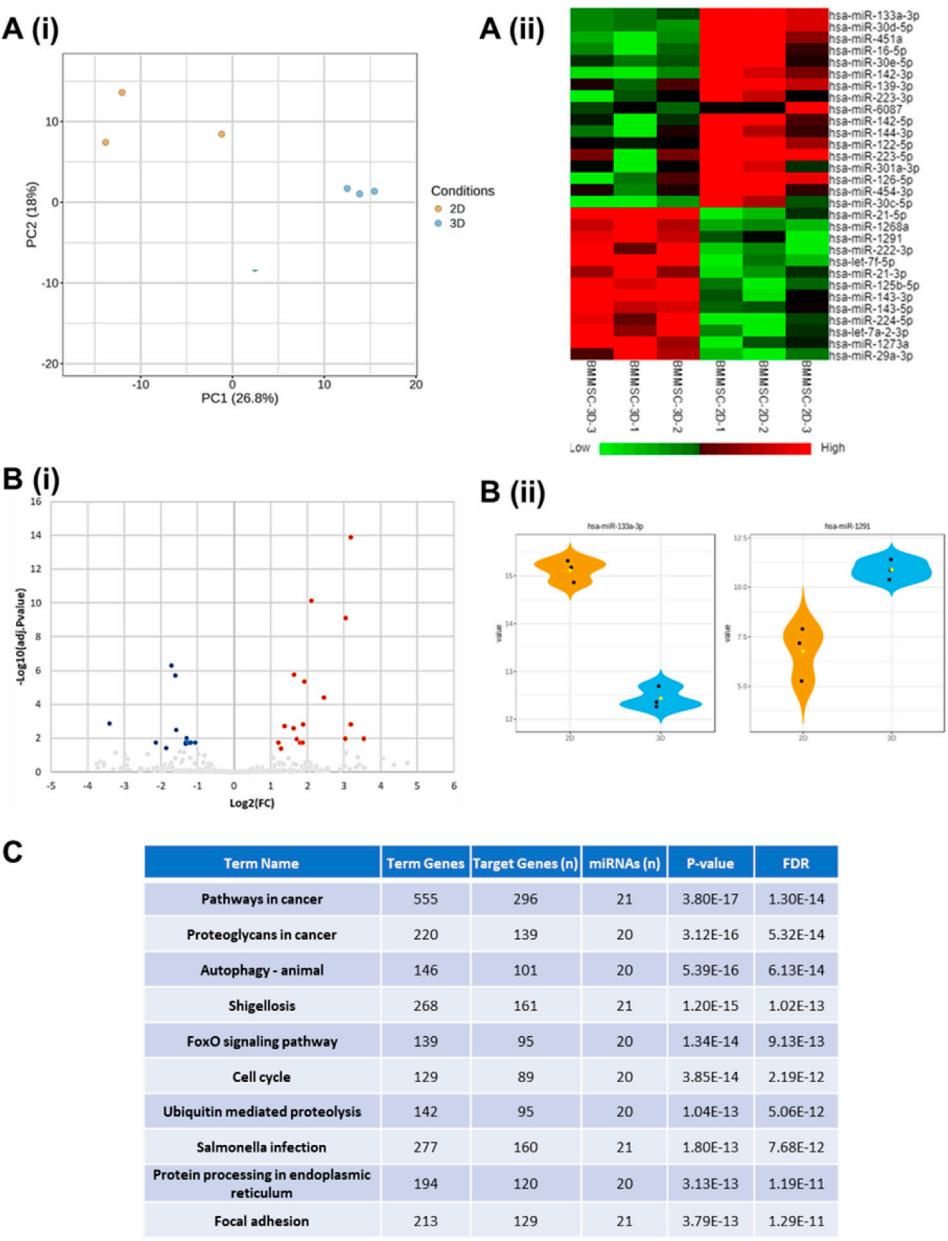
miRNA sequencing of EVs from 3D vs. 2D hMSCs for cargo analysis. Principal component analysis (PCA) plots of 3D vs. 2D hMSC-derived EVs in triplicate **(Ai)**. Heatmap illustration of differentially expressed genes (DEGs) from miRNA sequencing **(Aii)**. Volcano plot **(Bi)**. Violin plots expressing differential expression of hsa-miR-133a-3p and hsa-miR-1291 between hMSC EVs derived from 2D and 3D culture conditions **(Bii)**. KEGG pathway enrichment analysis of differentially expressed miRNAs **(C)**. 3D culture is from 40 rpm.

miRNA cargo for the EVs of 3D BM-hMSCs was performed in comparison to the EVs of 2D cells in triplicate using next-generation miRNA sequencing. Principal component analysis (PCA) revealed clear separation of the two groups with PC1 (23.5%) accounting for most of the variance, indicating that culture dimensionality strongly influences EV miRNA composition (Figure 6Ai). This is further supported by the heatmap (Figure 6Aii), which highlights distinct gene expression profiles between 2D and 3D hMSC-derived EVs. Notably, the differential miRNA expression levels in EVs of 2D and 3D cultures indicate fundamental differences in metabolic and signaling environments.

The volcano plot further illustrates differential gene expression between EVs of 3D versus 2D cultures, with significantly upregulated differentially expressed genes (DEGs) (17) within 3D hMSC EVs highlighted in red, significantly downregulated DEGs (13) within 2D hMSC EVs in blue, and non-significant DEGs (347) in gray (Figure 6Bi). Violin plots provide a detailed visualization of specific miRNA expression changes, focusing on hsa-miR-133a-3p and hsa-miR-1291 in EVs derived from both culture conditions (Figure 6Bii). hsa-miR-133a-3p is significantly upregulated in 2D hMSC EVs, where it is closely linked to the regulation of aerobic metabolism through the activation of the PKM2 enzyme (Alfardus et al., 2017). In contrast, hsa-miR-1291 is upregulated in 3D hMSC EVs, potentially reflecting a metabolic shift that supports the maintenance of a non-proliferative state in hMSC aggregates.

Lastly, the Kyoto Encyclopedia of Genes and Genomes (KEGG) pathway analysis highlights the statistically significant biological pathways (3D/2D) most influenced by the specific miRNA data, identified by the intensity of the blue coloring (Supplementary Figure S7). A detailed KEGG enrichment table summarizing the top pathways - including term genes, target genes, associated miRNAs, P-values, and false discovery rate values (Figure 6C). KEGG analysis identified signaling pathways relevant to EV biogenesis, metabolism, and neural modulation (Table 1). Particular miRNAs, such as hsa-miR-29a-3p, hsa-21-5p, hsa-133a-3p, have been identified as most influential in signal transduction regarding the Janus kinase signal transducer and activator of transcription (Jak STAT), phosphatidylinositol 3-kinase/protein kinase B (PI3K/Akt), ECM receptor interaction, vascular endothelial growth factor (VEGF), calcium, actin cytoskeleton, and axon guidance signaling pathways.

**TABLE 1:** miRNAs that intersect multiple regenerative and immunomodulatory pathways, underscoring their relevance in EV-mediated therapeutic strategies. Signaling pathways categorized by function (i.e., Pro-regenerative/proliferative signaling, inflammation and immune modulation, neural repair and guidance, and ECM and structural remodeling, respectively).

	Biological signaling pathways													
miRNA	P13k-Akt	VEGF	RAS	TGF-B	HIF-1	NF-kB	NOD	Jak-STAT	RIG-1-like receptor	Axon guidance	Calcium	Actin cytoskeleton	ECM-receptor	Wnt
miR-29a-3p														
miR-451a														
miR-223-3p														
miR-21-5p														
miR-125b-5p														
miR-224-5p														
miR-16-5p														
miR-133a-3p														
miR-143-3p														
miR-1268a														
miR-142-5p														
miR-30c-5p														

The shaded box indicates the involvement of miRNAs in this pathway.

### 3.5 Evaluation of EVs in inflammation of schwann cell functional assay *in vitro*

**FIGURE 7:**
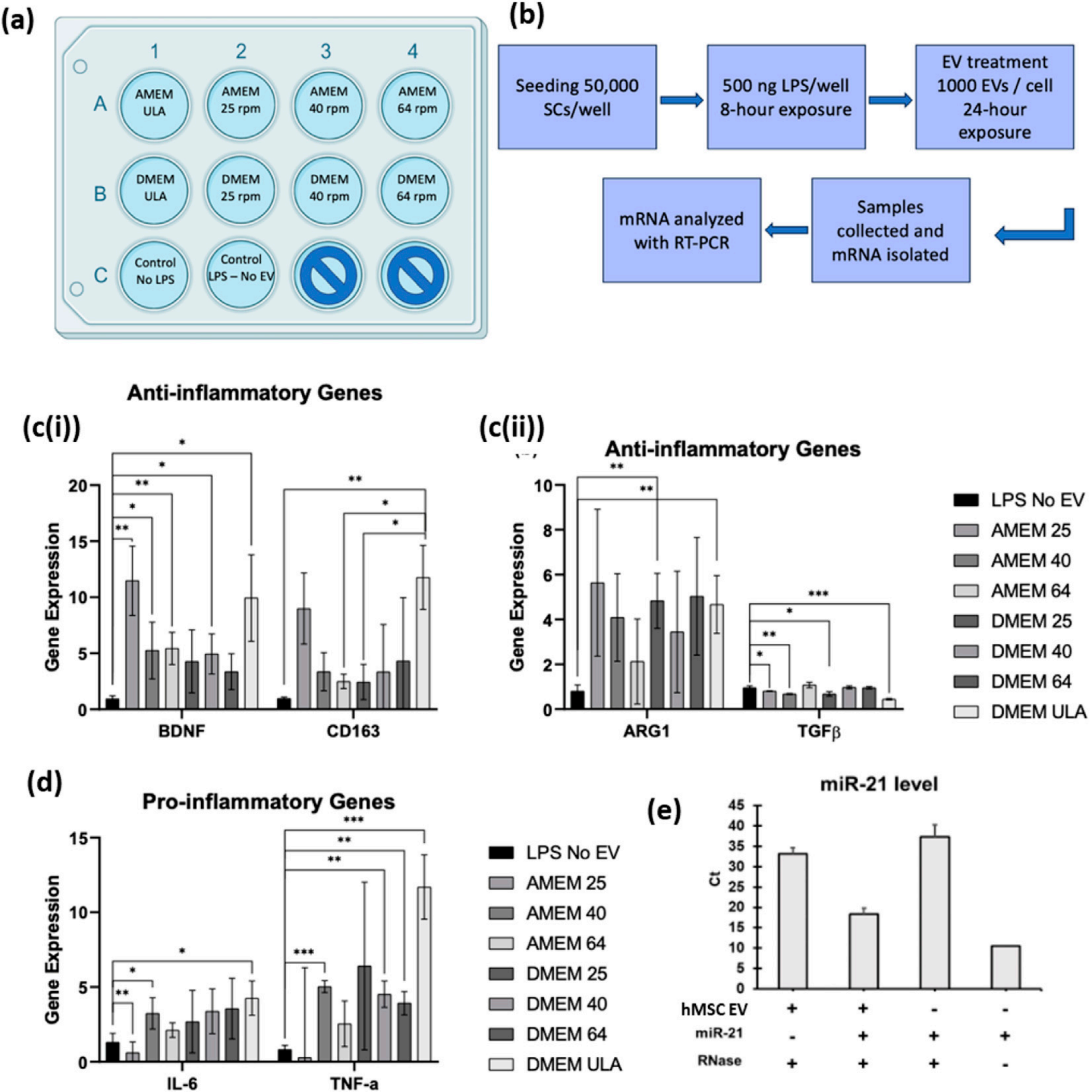
Evaluation of EVs in inflammation of Schwann cell functional assay *in vitro*. Experimental design of SCs treated with EVs from each experimental condition **(a)** and methodology to complete experiment **(b)**. Anti-inflammatory **(ci and cii)** and pro-inflammatory **(d)** gene expression of SCs treated with EVs determined by qRT-PCR. N = 3 (biological replicates from representative bioreactor run). *P < 0.05; **P < 0.01; ***P < 0.001. Loading miR-21 into the hMSC EVs. Detection of loaded miR-21 in hMSC-derived EVs following RNase treatment **(e)**.

To evaluate the therapeutic potential of 3D BM-hMSC-derived EVs from each experimental condition, hSCs were pre-treated with LPS for 8 h to activate NF-kB signaling (Figure 7a). Immunostaining confirmed nuclear translocation of the p65 subunit, indicative of pro-inflammatory gene activation (Supplementary Figure S8) (Hobbs et al., 2021). After inducing inflammation, SCs were treated at a dose of 1,000 EVs per cell for 24 h. This dose was selected based on previous studies and the EV protein per kg of body weight per dose within preclinical animal dosing guidelines (Liu et al., 2024; Gupta et al., 2021). Following treatment, SCs were harvested, and total mRNA was extracted and analyzed via qRT-PCR to assess changes in pro- and anti-inflammatory gene expression (Figure 7b). All experimental groups were included in the analysis, except for the ULA condition within the αMEM media group, which yielded insufficient mRNA for characterization.

Within all experimental conditions, the expression of the anti-inflammatory genes, including brain-derived neurotrophic factor (*BDNF*) (5–10 fold), *CD163* (3–10 fold), and *ARG1* (2-5 fold) were generally elevated compared to the untreated LPS-only control (Figure 7c). *BDNF* expression was significantly increased in most conditions, with αMEM 25 showing the highest level of expression. *CD163* expression was significantly upregulated with EVs derived from αMEM 25, αMEM 64, and DMEM ULA, while *ARG1* was only significant in DMEM 25 and DMEM ULA conditions. *TGFβ* gene expression showed minimal change across conditions.

Pro-inflammatory genes, *IL-6* and *TNF-α*, showed variable responses. *IL-6* and *TNF-α* were reduced in αMEM 25 compared to the LPS-only control, whereas other conditions showed inconsistent changes. Given the variability, no clear trend could be established for TNF-α across shear stress conditions (Figure 7d). Interestingly, the LPS-stimulated SCs treated with the BM-MSC-derived EVs resulted in an upregulation of both anti-inflammatory genes and pro-inflammatory genes across several experimental conditions. These results highlight potential short-term modulatory effects of EV cargo on LPS-induced inflammation, which warrants further investigation in longer-term studies.

Hsa-miR-21-5p has been shown to possess neuroprotective effects in spinal cord injuries, through the reduction of neural inflammation, and promotion of neurite outgrowth through the regulation of programmed cell death protein 4 (PDCD4) (Yuan et al., 2001; Jiang et al., 2021; Kang et al., 2019). As shown in Figure 6Aii, miR-21-5p level is higher in 3D culture condition than 2D. To assess the therapeutic potential of miRNA cargo delivered via EVs, an RNase protection assay was performed to evaluate the stability of miRNAs encapsulated within BM-hMSC-derived EVs (Figure 7e). After RNase digestion, qRT-PCR targeting miR-21 was performed, and the cycle threshold (Ct) values from different groups were compared, where lower Ct values correspond to higher levels of detectable miR-21. The miR-21 only groups with or without RNase treatment (group 3 and 4) supports the effectiveness of removal of free or un-bounded small RNA oligos. hMSC-EVs loaded with synthesized miR-21 oligo (group 2) was compared with hMSC-EV group, and the miR-21 level in loaded EVs was about 28000-fold higher than the original level from hMSC EVs. To be comparable, hMSC-EVs without mixing with miR-21 was also electroporated and these two groups were incubated with RNase A to remove free miR-21 oligos. The result shows that electroporation protocol can successfully introduce miR oligos into the EVs. Following RNase A digestion, the persistence of miR-21 signal suggests that the oligos were largely protected within EVs rather than freely bound externally. Together, these results highlight the ability of EVs to stably deliver functional miRNAs, underscoring their therapeutic potential as a cell-free treatment for neural inflammation.

## 4 Discussion

### 4.1 Influence of VWBR culture system on hMSC aggregation

While aggregate culture of hMSCs offers advantages by better mimicking natural tissue environments than 2D culture, it is also associated with a reduced proliferation rate. The 3D culture environment enhances the expression of therapeutic, immunosuppressive, and anti-inflammatory factors, yet concurrently limits hMSC proliferation through cell cycle arrest (Yuan et al., 2001; Burand et al., 2025). Unlike hMSC 2D culture, hMSC aggregation undergoes distinct metabolic processes that influence the cellular phenotype (Burand et al., 2025).

In this study, the total cell count progressively declined, which can be attributed to the influence of shear stress on aggregate formation. Cells that successfully integrated into aggregates were protected through cell-to-cell adhesion and exhibited condensation behavior with reduced proliferation, whereas cells excluded from aggregates were lost (Yuan et al., 2001; Petry et al., 2018; Jeske et al., 2022; Takebe et al., 2025). Thus, two parallel processes occur: (i) cells within the aggregate undergo condensation behavior and cell cycle arrest as a survival and preservation strategy (Bijonowski et al., 2020), while (ii) shed or excluded cells fail to survive, leading to the declined cell number. This reflects a shift from proliferation toward a compact, metabolically active state, where cells maintain high viability and concentrate cellular material.

Aggregation kinetics studies provide additional insight into these processes. Tsai et al. demonstrated that hMSC aggregation in dynamic suspension depends on sequential events of collision, adhesion, and actin-myosin-mediated compaction, all of which are shaped by hydrodynamic conditions (Tsai et al., 2025; Tsai et al., 2024). Shear stress and cell adhesion molecules such as cadherins and collagen play distinct roles in aggregate formation (Tsai et al., 2024; Tsai et al., 2025). Cadherins are critical for strengthening early cell-to-cell contacts, while collagen and other extracellular matrix proteins modulate the formation of smaller clusters into larger spheroids (Tsai et al., 2024). Importantly, higher shear stress limited the opportunity for stable adhesion, resulting in smaller and more uniform aggregates, whereas inhibition of cadherin-mediated adhesion disrupted aggregate growth altogether (Tsai et al., 2025). These findings support our interpretation that cells unable to establish stable adhesive interactions under shear stress are shed into the medium and ultimately lost, likely through anoikis.

### 4.2 Influence on cellular metabolism and EV biogenesis

Our study hypothesizes that glycolytic shift in 3D aggregates reflects both oxygen diffusion and the requirement to preserve stemness, as glycolysis allows hMSCs to maintain viability under hypoxic stress while reducing cellular reliance on oxidative phosphorylation. In addition, glycolysis-associated miRNAs (miR-133a-3p, miR-143-3p, and miR144-3p) may further reinforce this metabolic adaptation by regulating pathways such as PI3K-Akt and mTOR, ultimately linking energy metabolism with enhanced EV biogenesis.

hMSC aggregates undergo a metabolic shift toward glycolysis through mitochondrial respiration, which is driven by oxygen gradient in order to maintain hMSC stemness, viability, and proliferation (Burand et al., 2025; Rybkowska et al., 2025). Within culture environment, the internal cells of the 3D spheroid experience hypoxic microenvironment due to the nutrients diffusion (Kamprom et al., 2024). hMSC aggregates exhibit increased reactive oxygen species (ROS) production and decreased intracellular lactate production (Burand et al., 2025). Furthermore, glycolytic gene expression that serves as evidence of aerobic glycolysis, such as *G6PDH, LDHA, PDHA, PFKM*, and *PFKFB3*, increased expression, as well as the relative protein level of PFK-1, PFK-2, and G6PDH within 3D aggregates compared to 2D cultures (Burand et al., 2025).

Glucose metabolism through glycolysis yields pyruvate, which is reduced by lactate dehydrogenase (LDH) to produce lactate, while NADH simultaneously oxidized to NAD^+^. Under glutamine-rich conditions, glutaminase converts glutamine to alpha-ketoglutarate (αKG), producing ammonia as a byproduct. The expression of *PDK1* correlates with the regulation of pyruvate dehydrogenase complex, which phosphorylates pyruvate to acetyl-CoA within the mitochondria to produce energy (Arnold and Finley, 2022). *HK2* is closely related to the phosphorylation of glucose toward the production of ATP, as well as the promotion of cellular survival by regulating mitochondrial pro-survival factors and inhibiting mammalian target of rapamycin complex 1 (mTORC1) to promote autophagy (Guo et al., 2023). LDHA and PKM2 are critical enzymes in glycolysis, and the upregulated expression is evidence of glucose uptake resulting in the by-product secretion of lactate (Hong et al., 2020). The upregulation in *G6PD* gene expression demonstrates increased aerobic glycolysis and supports the reoxidation of NADH to NAD^+^ (Arnold and Finley, 2022). This process is known as the tricarboxylic acid cycle, which regulates the oxidation of cellular nutrients within the metabolic pathway to generate energy (Arnold and Finley, 2022). Regarding PPP activation, qRT-PCR data, alongside increased glutamine consumption in VWBR, suggest enhanced NADPH production–a hallmark of cells redirecting glucose through the PPP in response to oxidative stress (TeSlaa et al., 2023). It is postulated that this metabolic plasticity may be correlated to EV biogenesis. Further study may use small molecules to disturb the metabolic pathways and evaluate the influence on EV biogenesis to confirm this correlation.

Various other studies show the cause-and-effect relationship between nutrient metabolism and the induction of intrinsic signaling pathways that regulate hMSC function, gene expression, and degree of differentiation (Hong et al., 2020; Mohammadalipour et al., 2020; Sun et al., 2020). The induction of various pathways has been attributed to the role of miRNAs regulating receptor tyrosine kinases (RTKs) on the cellular surface that can influence the regulation of glycolytic metabolism, as well as cell death, proliferation, and angiogenesis (Alfardus et al., 2017). With the shift toward aerobic glycolysis, the upregulation of miR-144-3p targets GLUT1, leading to a reduction in anaerobic glycolysis through the downregulation of glucose uptake and lactate production (Morais et al., 2021). Liu et al. demonstrated the influence of miR-144 on aerobic metabolism (Liu et al., 2016). This process is evident by the complimentary relationship between the increased expression of GLUT1 and increased glucose uptake and lactate production (Liu et al., 2016). The metabolic shift towards aerobic metabolism results in enhanced proliferation of the cancerous cells. This pattern is consistent with the decreased shift in hMSC aggregate cellular proliferation and increased expression of miR-144-3p in 3D culture conditions.

Similarly, the pentose phosphate pathway is crucial in regulating cellular oxidative stress by generating NADPH, a key reducing agent that supports the regeneration of antioxidants (TeSlaa et al., 2023). In addition, PPP produces ribose 5-phosphate (R5P), which is essential for nucleic acid biosynthesis, and recycles carbon back into glycolysis to maintain metabolism (Meyer et al., 2018). Given the high glucose medium formulation compared to the αMEM/FBS cultures, DMEM/F12/B27 cultures may have different metabolic pathways. The shift between glycolysis and oxidative phosphorylation may need further investigation. Differentiated hMSCs have shown an enhanced capacity for both carbohydrate metabolism and mitochondrial activity, with increased LDH activity indicating reliance on lactate as an energy source and engagement of aerobic glycolysis (Meyer et al., 2018). These findings underscore the critical role of PPP activity in balancing biosynthetic needs and redox homeostasis, processes that directly influence stem cell fate and EV biogenesis (Riegger et al., 2023; Dellar et al., 2024). In this study, increased PPP activation–particularly in αMEM/FBS–suggests a shift toward enhanced antioxidant defense, which could influence EV yield and immunomodulatory function. Nevertheless, the data indicate that 3D dynamic aggregate culture alters metabolic activity and potentially promotes EV biogenesis compared to static culture. Notably, the gene expression findings were consistent with metabolite data, indicating the possible correlation between dynamic culture, metabolic adaptation, and EV biogenesis.

### 4.3 Influence on hMSC EV miRNA cargo profile

Unlike other 3D systems where hypoxic cores form, hMSC aggregates avoid severe diffusion limitations (Bijonowski et al., 2020) and adapt the aggregate microenvironment by reducing oxygen uptake and shifting metabolism towards glycolysis (Yuan et al., 2001; Bijonowski et al., 2020; Liu et al., 2019; Liu et al., 2020). This adaptation is consistent with EV cargo profiles, where upregulation of miR-1291 and miR-143-3p from 3D cultures supports a role in the G0/G1 phase of mitosis (Cai et al., 2021; Wang et al., 2020). Furthermore, the EV-cargo miRNA profiles confirm that 3D dynamic aggregate culture significantly modulates intracellular signaling in hMSCs by altering the composition of EV cargo. The upregulation of miR-29a-3p, miR-451a, miR-224-5p, miR-16-5p, miR-133a-3p, and miR-143-3p reflects a combination that enhances EV biogenesis, promotes metabolic reprogramming, and supports immunomodulatory behavior characteristics of 3D cultures. miR-29a-3p is a key regulator in ECM remodeling, axon guidance, and PI3K-Akt and Wnt signaling pathways, contributing to neural regeneration and cellular plasticity (Kang et al., 2024; Dalgaard et al., 2022; Kwon et al., 2019; Gao et al., 2019). miR-224-5p and miR-16-5p are associated with the suppression of proliferative signaling, inflammation control, and stress adaptation through key regulatory pathways, such as NF-kB, TGF-B, and Jak-STAT signaling, aligning with a non-proliferative, anti-inflammatory phenotype observed in 3D aggregates (Zhang et al., 2022; Tian et al., 2025; Xu et al., 2019). miR-451a helps regulate mitochondrial function, and manage oxidative and cellular stress adaptation, promoting cellular resilience (Scrimgeour et al., 2019; Shao et al., 2025). miR-133a-3p and miR-143-3p may contribute toward modulating glucose flux between aerobic and anaerobic glycolysis, likely influenced by the culture medium composition, which targets PKM2 to influence PI3K-Akt signaling cascades (Guo et al., 2021; Abolhasani et al., 2025).

Collectively, these miRNAs are enriched in EVs, highlighting the ability to target signaling networks central to cell survival, immune regulation, metabolic stability, ECM dynamics, and neuro-regeneration. Consequently, the EV cargo derived from 3D hMSC cultures is enriched with molecules associated with regenerative and immunomodulatory functions, suggesting its potential to modulate neuroinflammatory responses in short-term models and serving as a foundation for future studies exploring longer-term therapeutic applications. Together these findings indicate that aggregate culture showed adaptive metabolic preservation and produced EVs with cargo enriched for pathways that promote survival and immunomodulatory activities.

### 4.4 Effects of hMSC EVs on neural modulation

Finally, aggregate culture alters hMSC immunomodulatory behavior. Burand et al. demonstrated that hMSC aggregates increase expression of immune response factors prostaglandin E2 (PGE2), tumor necrosis factor-stimulated gene-6 (TSG-6), IL-1α/β, and TGF-B1, while downregulating CD73 and indoleamine 2,3- dioxygenase (IDO) (Burand et al., 2025). As an aggregate, hMSCs decrease the ability to inhibit T-cell proliferation, evident by the lowered expression of CD73, a regulatory immunosuppressive factor, and the increase of T-cell expression compared to 2D culture (Burand et al., 2025). Aggregates influence the plasticity of macrophages, allowing the increased binding of PGE2 to the macrophage EP2 and EP4 receptors, shifting the polarity from inflammatory (M1) to anti-inflammatory (M2) phenotype (Kamprom et al., 2024). With the upregulation of these anti-inflammatory and immunomodulatory molecules, hMSC aggregates support the secretion of beneficial paracrine factors and EVs (Kamprom et al., 2024).

In this study, the SC functional assay evaluated the effectiveness of BM-hMSC-derived EVs on inflamed SCs exposed to LPS. LPS stimulation activates the NF-kB pathway, promoting nuclear translocation of the p65 subunit and the subsequent transcription of inflammatory genes. A time-course optimization study determined that 500 ng/mL LPS for 8 h resulted in maximal nuclear translocation of NF-kB, as confirmed by immunostaining. Interestingly, RT-PCR analysis revealed that the LPS-only group (LPS No EV) exhibited unexpectedly low expression of inflammatory markers, with the exception of the αMEM 25 condition. This discrepancy may be attributed to negative feedback regulation or desensitization of cytokine signaling after prolonged LPS exposure (Hobbs et al., 2021). Since mRNA was harvested approximately 32 h after initial LPS treatment (following 24 h of EV exposure), it is likely that the immune response in the LPS No EV group had already begun to resolve, while EV-treated groups were still actively modulating inflammation.

EVs derived from BM-hMSCs cultured in αMEM and DMEM conditions excreted anti-inflammatory effects. This was demonstrated by the concurrent upregulation of anti-inflammatory markers (e.g., *CD163*, *ARG1*) in the treated SCs. The EVs may contain miRNA cargo relevant to neural modulation and peripheral nerve regeneration. Notably, the significant increase in expression of miR-29a-3p, miR-133a-3p, and miR-143-3p in dynamically cultured BM-hMSCs is associated with the activation of signaling pathways such as axon guidance, ECM-receptor interaction, and actin cytoskeleton remodeling. These pathways play essential roles in synapse formation, growth cone dynamics, and ECM reconstruction, which are processes critical in nerve repair (Leite et al., 2021; Xu et al., 2024). Additionally, activation of PI3K-Akt, Wnt, and VEGF pathways supports synaptic plasticity, SC remyelination, and neovascularization through the nutrient and oxygen delivery to the damaged site (Narvaes and Furini, 2022; Gaesser and Fyffe-Maricich, 2016; Pan et al., 2013).

Previous reports indicate that EVs can simultaneously activate both pro- and anti-inflammatory pathways depending on the recipient cell physiological status and surrounding environment (Zhou and Bréchard, 2022; Hezel et al., 2017). Zhou et al., utilized neutrophil-derived EVs to demonstrate a dual response, promoting pro-inflammatory signals while upregulating anti-inflammatory mediators. This reflects the complex immune-modulatory role the EVs possess that fine tune the immune response rather than exclusively suppressing inflammation (Zhou and Bréchard, 2022). Overall, the data indicate that EVs derived from VWBR cultures can influence pro- and anti-inflammatory gene expression in SCs over the short-term, though the responses are context-dependent. These findings support the conclusion that dynamic culture conditions promote the secretion of immunomodulatory EV cargo capable of attenuating inflammation and promoting peripheral nerve repair.

### 4.5 Experimental limitations

While the results suggest enhanced EV biogenesis under specific metabolic and culture conditions, several limitations regarding EV characterization should be noted. NTA was performed in scatter mode, which makes it difficult to distinguish EVs from co-isolated particles, such as lipoproteins, apoptotic bodies, and cellular debris. Future studies could address this by utilizing fluorescent-mode NTA to improve specificity. For ESCRT, Glycolytic, and PPP gene expression analysis, the RNA yields from the 25 rpm condition of αMEM/FBS groups were insufficient for downstream RT-PCR analysis. Based on our previous study (Jeske et al., 2023) and Figure 5 in this study, there was no consistent trends of increased EV secretion in the range of 25–64 rpm agitation. But the presence of the agitation consistently increased EV secretion compared to static culture conditions. Similarly, for the DMEM/F12/B27 condition, RNA input was also not sufficient to complete RT-PCR analysis. In the case of the ULA control, the overall lower seeding density led to reduced aggregate viability and lower final cell yield. As a result, not all ULA samples produced enough material for downstream analysis. While these missing data points limit direct comparisons across all culture conditions, the datasets that were successfully obtained provide consistent and biologically meaningful treads across independent runs. In addition, the seeding density may be increased and the intermittent agitation regime can be designed to promote the hMSC aggregation within the first 24 h.

## 5 Conclusion

This study demonstrates that 3D hMSC aggregates within the VWBRs can efficiently produce EVs with enhanced yield and therapeutic cargo compared to static controls. VWBR culture supported the formation of hMSC aggregates, where cells within aggregates maintained high viability but underwent condensation and cell cycle arrest, while non-aggregated cells were lost, likely through anoikis. These dynamics, shaped by shear stress, influenced both survival and EV secretion. Metabolic and miRNA analyses revealed that VWBR conditions promoted the shift from oxidative phosphorylation toward glycolysis and increased PPP activity. Furthermore, the upregulation of miR-1291 and miR-143-3p supports therapeutic roles in regulation and metabolic reprogramming. Functionally, EVs from VWBR were associated with increased expression of anti-inflammatory genes (*BDNF, CD163*, and *ARG1*) and selective suppression of pro-inflammatory markers (*IL-6*, *TNF-α*) in SCs exposed to LPS. Notably, both pro- and anti-inflammatory genes were upregulated, reflecting context-dependent EV effects. Overall, VWBR culture emerges as a scalable strategy for generating hMSC-EVs with high yield and functionally relevant cargo. By influencing aggregate behavior, metabolism, and cell cycle state, VWBRs create a microenvironment that preserves viable, functionally active hMSCs. These findings provide insight for promoting EV biogenesis in dynamic culture systems and reinforce the translational promise of hMSC-EVs for regenerative medicine.

## Data Availability

The original contributions presented in the study are publicly available. This data can be found here: https://www.ncbi.nlm.nih.gov/geo/query/acc.cgi?acc=GSE316471.
