# Supplementary material for: Engineering human bone marrow-derived mesenchymal stromal cell aggregates for enhanced extracellular vesicle secretion in a vertical-wheel bioreactor
Source: Front Bioeng Biotechnol. 2026 Jan 16;13:1664302. doi: 10.3389/fbioe.2025.1664302 (PMC12857299; doi:10.3389/fbioe.2025.1664302)
Supplement: Supplementary file 1 [file Supplementaryfile1.docx]

**Supplementary Materials**

**Engineering Human Bone Marrow-derived Mesenchymal Stromal Cell Aggregates for Enhanced Extracellular Vesicle Secretion in a Vertical-Wheel Bioreactor**

Danyale Berry^1,2^, Breana Boirie^3^, Mandip Singh^3^, Li Sun^4,5^,

Sunghoon Jung^6^, Yan Li^4, *^, Changchun Zeng^1,2, *^

1. Department of Industrial and Manufacturing Engineering, FAMU-FSU College of Engineering, Florida Agricultural and Mechanical University, Tallahassee, FL 32310, USA.

2. High Performance Materials Institute, FAMU-FSU College of Engineering, Florida State University, Tallahassee, FL 32310, USA.

3. College of Pharmacy and Pharmaceutical Sciences, Florida Agricultural and Mechanical University, Tallahassee, Fl, 32307, USA;

4. Department of Chemical and Biomedical Engineering, FAMU-FSU College of Engineering, Florida State University, Tallahassee, FL 32310, USA.

5. Department of Biomedical Sciences, College of Medicine, Florida State University, Tallahassee, Florida, USA

6. PBS Biotech Inc., Camarillo, CA 93012, USA.

**Supplementary Table S1. A list of antibodies.**

| **Markers** | **Primary Antibody** | **Origin/ Isotype** | **Supplier/ Cat#** | **Dilution** |
| --- | --- | --- | --- | --- |
| Exosomal markers | CD63 | Mouse monoclonal, IgG_1_ | Abcam; TS63 | 1:1000 |
|  | HRS | Rabbit IgG | Cell Signaling Technology, 15087 | 1:1000 |
|  | CD9 | Mouse monoclonal, IgG_1_ | Millipore; MM2/57 | 1:1000 |
|  | Calnexin | Mouse monoclonal, IgG_1_ | Santa Cruz, S-70 | 1:1000 |
| Inflammation marker | NF-KB P65 | Rabbit monoclonal IgG | Cell Signaling Technology, 8242S | Immunocytochemistry: 1:400 |
| Secondary | IRDye® 800CW | Goat anti-mouse IgG_1_ | LI-COR, 926-32350 | Western blot: 1:5,000 |
|  | IRDye® 800CW | Goat anti-Rabbit IgG (H + L) | LI-COR, 926-32211 | Western blot: 1:5,000 |
|  | Alexa Fluor™ 488 | Goat anti-Rabbit IgG (H + L) | Invitrogen, A-11008 | Immunocytochemistry and Flow cytometry: 1:400 |

**Supplementary Table S2. Primer information for qRT-PCR analysis.**

| **Gene name** | **Primer sequence (5’-3’)** |
| --- | --- |
| h-ACTB | GTACTCCGTGTGGATCGGCG |
|  | AAGCATTTGCGGTGGACGATGG |
| h-PDK1 | AAACAGGGGAGCTTTGTCTGG |
|  | CTGCCCATTCACATCCCTCTA |
| h-HK2 | TGGTGTAGCTCCTCTGCTGCT |
|  | TGTGGGCACCCTTTAGTGAAC |
| h-PKM2 | AAAAATGGATGCCCAGAGGAC |
|  | GAGTCGGCTTCAATGGAACAA |
| h-LDHA | CCTTGAGCCAGGTGGATGTTT |
|  | CACTGGATCCCAGGATGTGAC |
| h-G6PD | CTACCCGAGCCCAGCTACATT |
|  | TTCTGTTGGGCTGGAGTGAGT |
| h-6PGD | CCATGCCCTGTTTTACCACTG |
|  | AGGTGTGAGCCCCGAAGTAAT |
| h-TALDO1 | CTGTCATCAACCTGGGAAGGA |
|  | GGGCGAAGGAGAAGAGTAACG |
| h-TKTL1 | ACCTTGGGATTCTGTGTGCTG |
|  | CCTAACAAGCTTTCGCTGCTG |
| h-SMPD2 | GCCTGGGAGACTTTCTGAACC |
|  | AAGTGGTGTGCAGCTGGGTAG |
| h-SMPD3 | TTAAGAGACTCCAGGGCTGCTC |
|  | CGGGGATTGTCAAAAACAGTC |
| h-SRSF5(HRS) | CTTCTCGGATCGAGGCTTCTT |
|  | TCGAATCAACTGCGCTCATTA |
| h-STAM1 | CACTGGATTTTTGGGTTGCTC |
|  | GTGGAAAACATTTTTCGCATGA |
| h-TSG101 | CACCTGGTGGTCCATATCCTG |
|  | GATGGTGTCCTCGCTGATTGT |
| h-PDCD61P (ALIX) | TAAGTGCATCTGAGGGCCAAA |
|  | GGGGCCTCCTTTCCTAGTTTC |
| h-RAB27A | GCATGTTTCAGTTTTCAAGAACCA |
|  | AAAGGTGGCTTTTGTGTGTGC |
| h-RAB27B | TCCATGAAGCTGCTTGTCTCA |
|  | GTTGGGTCTCCACCCAGAAAT |
| h-IL-6 | ACCCCCAATAAATATAGGACTGGA |
|  | GGAGTTCATAGCTGGGCTCCT |
| h-BDNF | AATTTTGCTCCCCAGTGAAGG |
|  | GGCTCCCAACTTGACTTCTCC |
| h-CD163 | CCAGTCCCAAACACTGTCCT |
|  | ATGCCAGTGAGCTTCCCGTTCAGC |
| h-Arg (Arginase)-1 | GGCAAGGTGATGGAAGAAACA |
|  | GTGTGCCAGTAGCTGGTGTGA |
| h-TNF-a | CGTCGTAGCAAACCACCAAG |
|  | TTGAAGAGAACCTGGGAGTAGACA |
| h-TGF-β | GAAGAACTGCTGCGTGCGGC |
|  | CTGCGTGTCCAGGCTCCAAATG |

**Supplementary Table S3. EV production normalized to original cell count.** Summary of EV production. EV concentrations obtained by NTA on day 3 of culture. Due to the difference in cell number, across conditions, the data within the manuscript was normalized to EV/cell.

| **Condition** | **Total EVs (×10^6^)** |
| --- | --- |
| **αMEM ULA** | **0.53** |
| **αMEM 25 rpm** | **43.2** |
| **αMEM 40 rpm** | **37.4** |
| **αMEM 64 rpm** | **80.8** |
| **DMEM ULA** | **16.1** |
| **DMEM 25 rpm** | **106.0** |
| **DMEM 40 rpm** | **187.3** |
| **DMEM 64 rpm** | **310.9** |

**Supplementary Figure S1. Calnexin negative control marker to confirm cellular expression but absence in EVs.**

**
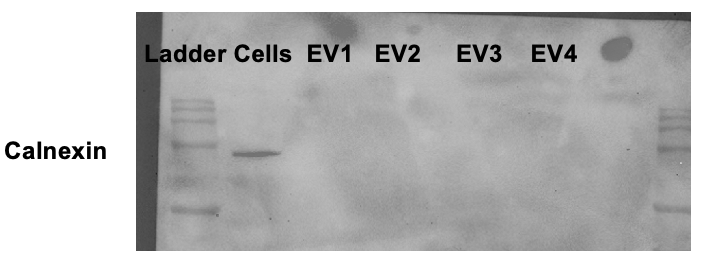
**

**Supplementary Figure S2. Establishing working cell bank of hMSCs in T flasks.** (A) Culture morphology over time of P+1. (B) Culture morphology over time of P+2. Scale bar of 4x: 500 μm. Scale bar of 10x: 200 μm. Scale bar of 20x: 100 μm.

**
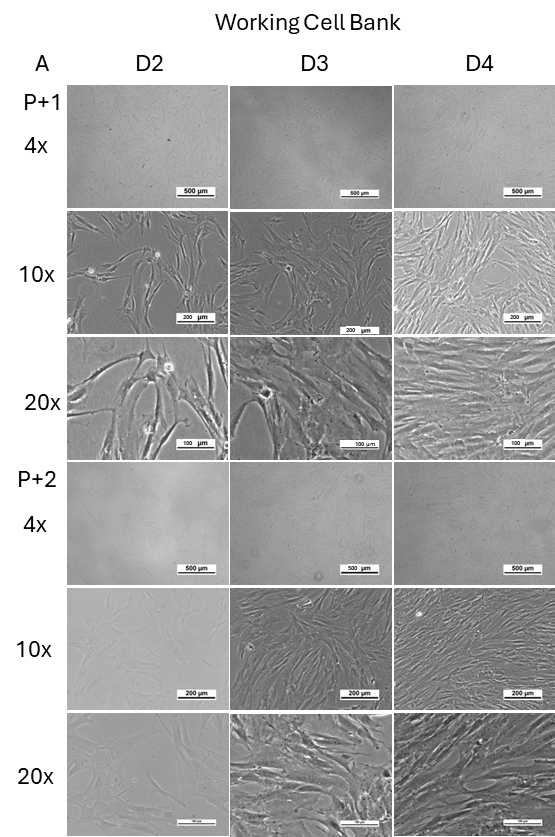
**

**Supplementary Figure S3. Experiment 1 Run 3. hMSC aggregates in VWRBs within αMEM/FBS media from 25 rpm, 40 rpm, and 64 rpm culture conditions.** (A) Culture morphology over time. Scale bar of 4x: 500 μm. Scale bar of 10x: 200 μm. (B) Total cells over time normalized to cells/mL. (C) pH kinetics over time. (D) Cellular viability over time.

**
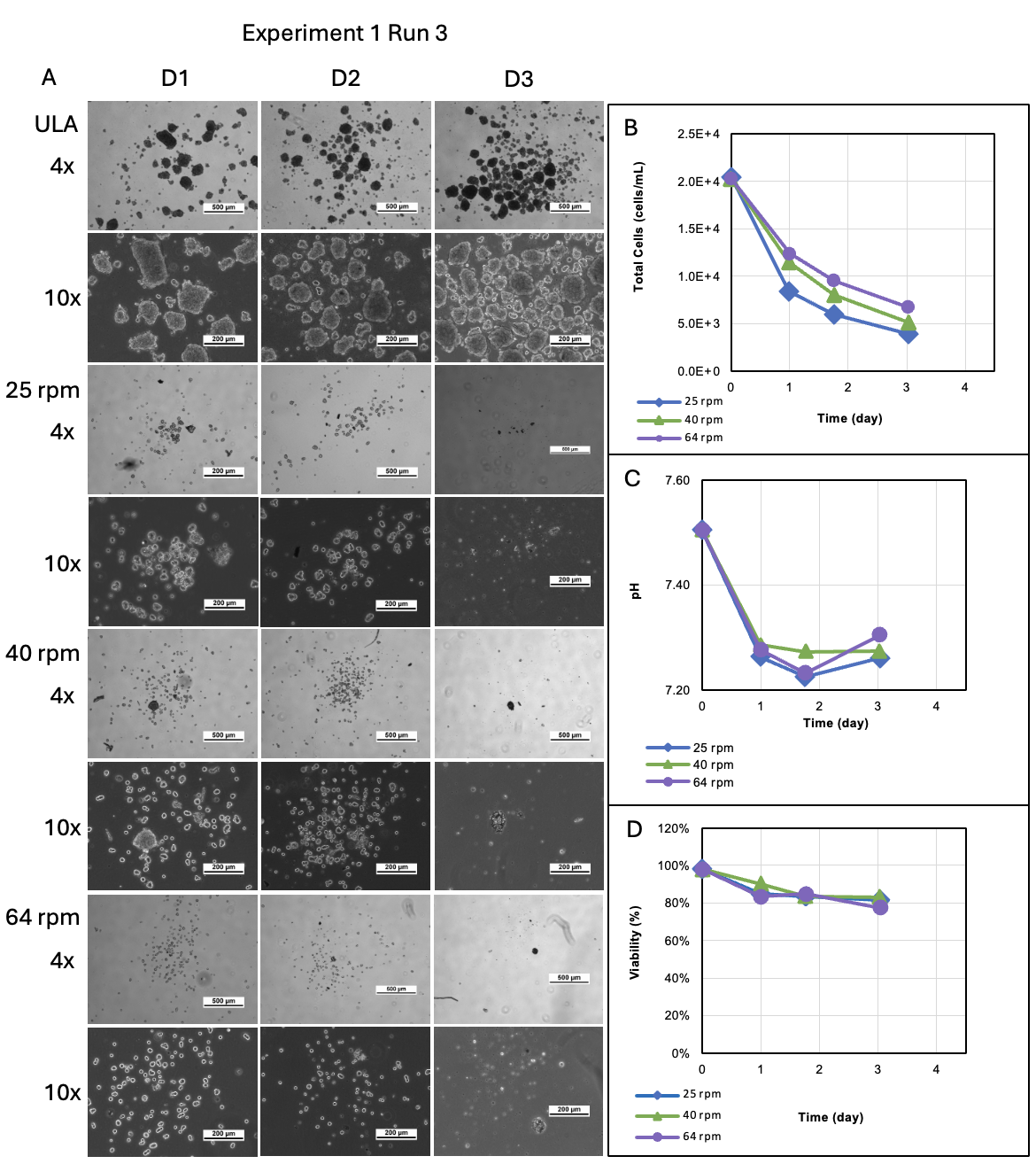
**

**Supplementary Figure S4. Experiment 2 Run 2. hMSC aggregates in VWRBs within DMEM/B27 media from 25 rpm, 40 rpm, and 64 rpm culture conditions.** (A) Culture morphology over time. Scale bar of 4x: 500 μm. Scale bar of 10x: 200 μm. (B) Total cells over time normalized to cells/mL. (C) pH kinetics over time. (D) Cellular viability over time.


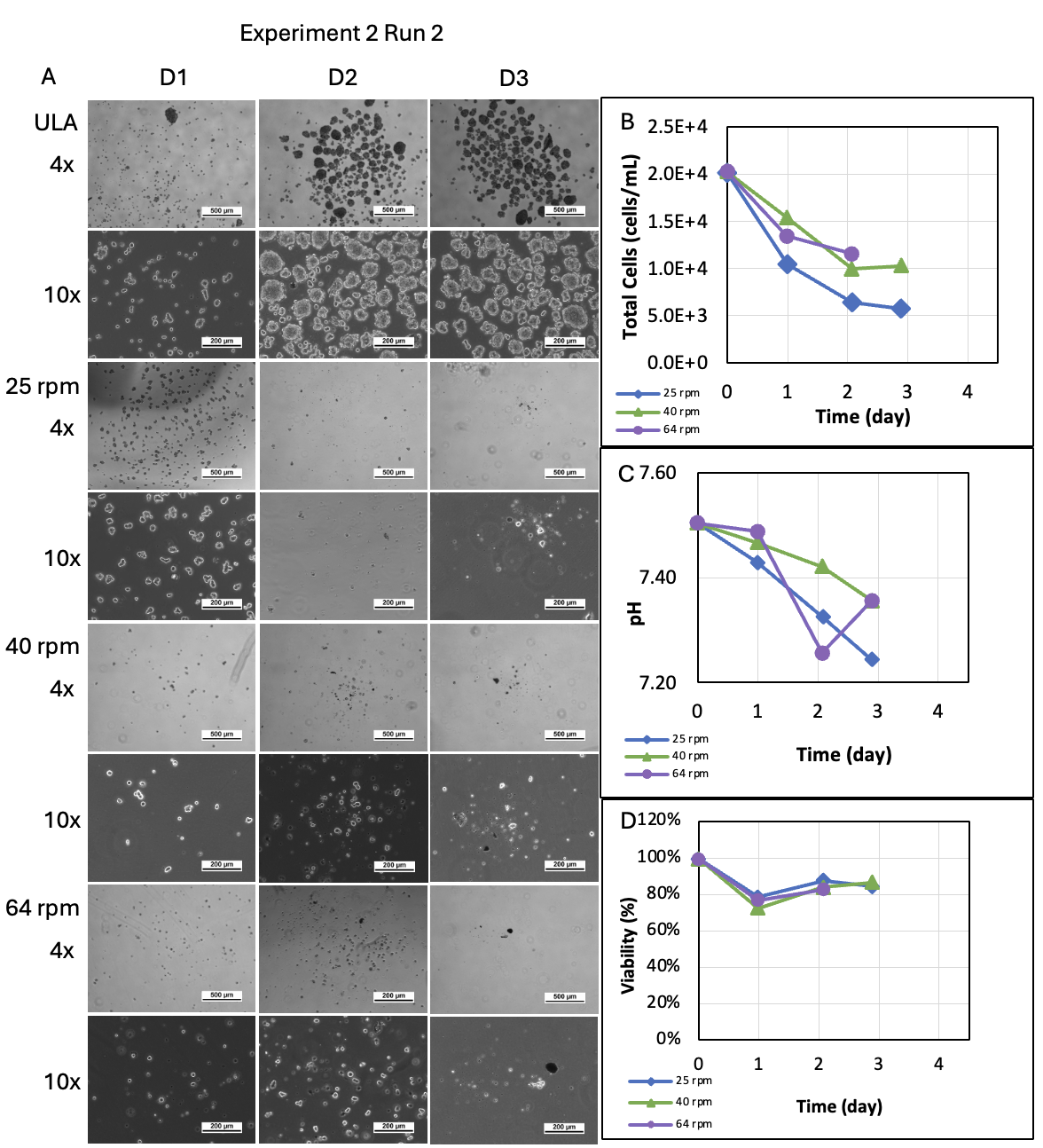


**Supplementary Figure S5. qRT-PCR analysis for mRNA expression of EV biogenesis markers in hBM-MSCs**. The comparison was performed for agitation speeds of 40 and 64 rpm to the static ULA control within DMEM-Run. (a) ESCRT independent EV biogenesis genes; (b) ESCRT dependent EV biogenesis genes. N=3, * indicates p<0.05 and ** indicates p<0.01.

**
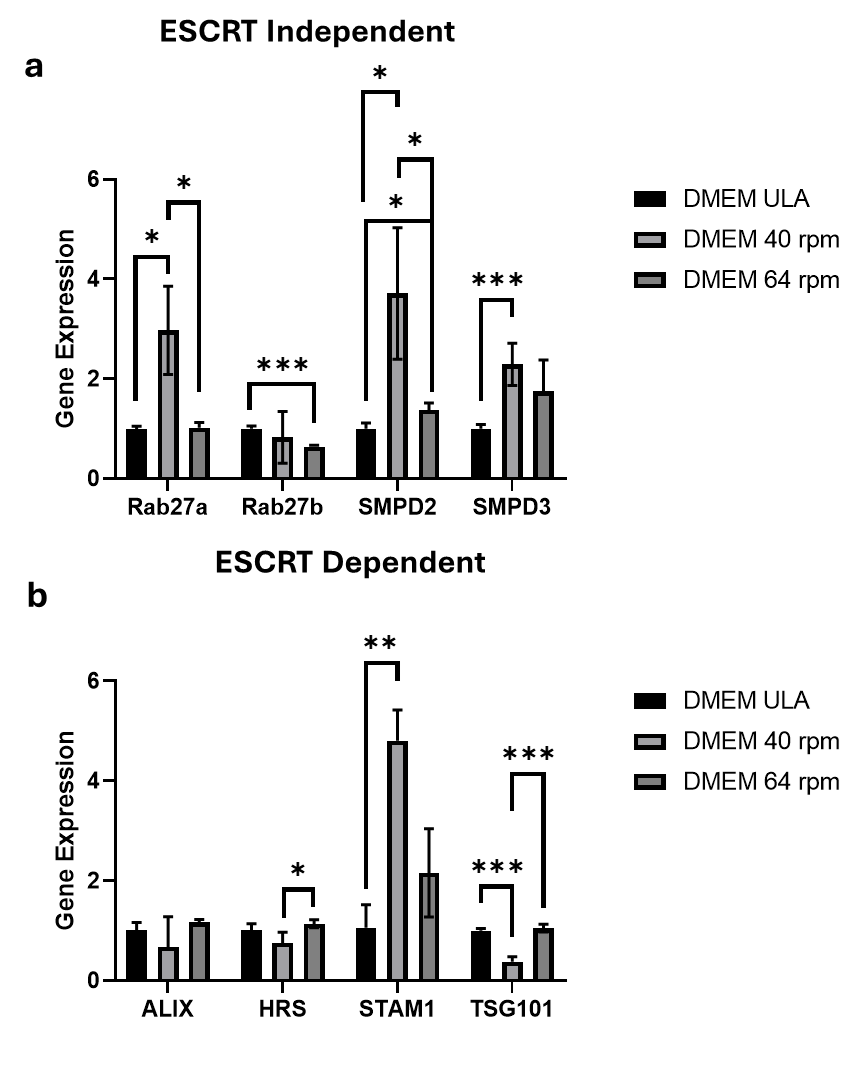
**

**Supplementary Figure S6. Representation of particle size distribution of the EVs measured by nanoparticle tracking analysis.** (A) EVs derived from experiment 1 run 1 within αMEM/FBS media. (B) EVs derived from Experiment 1 Run 2 within αMEM/FBS media. (C) EVs derived from experiment 2 run 1 within DMEM/B27 media. (D) EVs derived from Experiment 2 Run 2 within DMEM/B27 media.


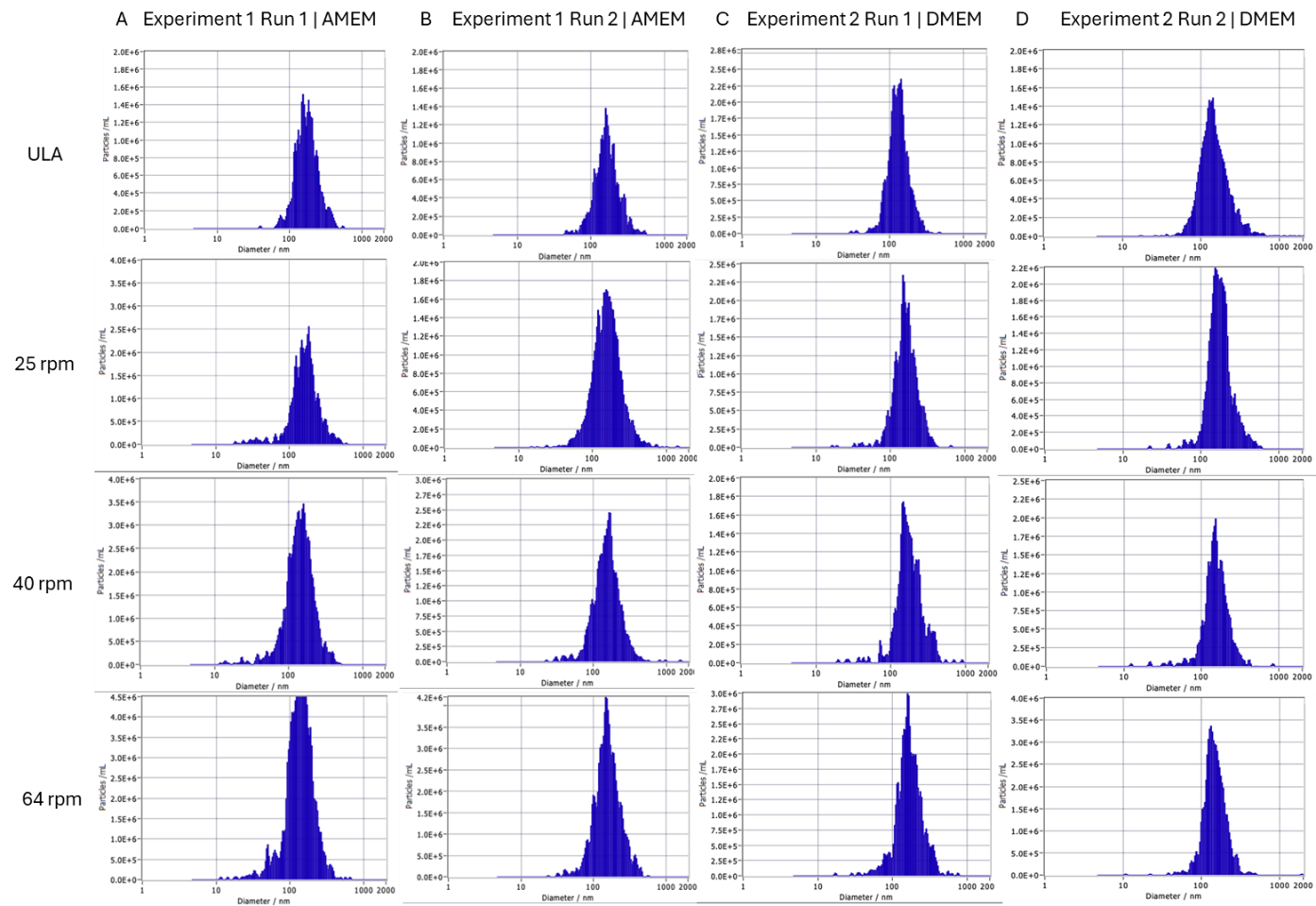


**Supplementary Figure S7. KEGG pathway enrichment graph.**


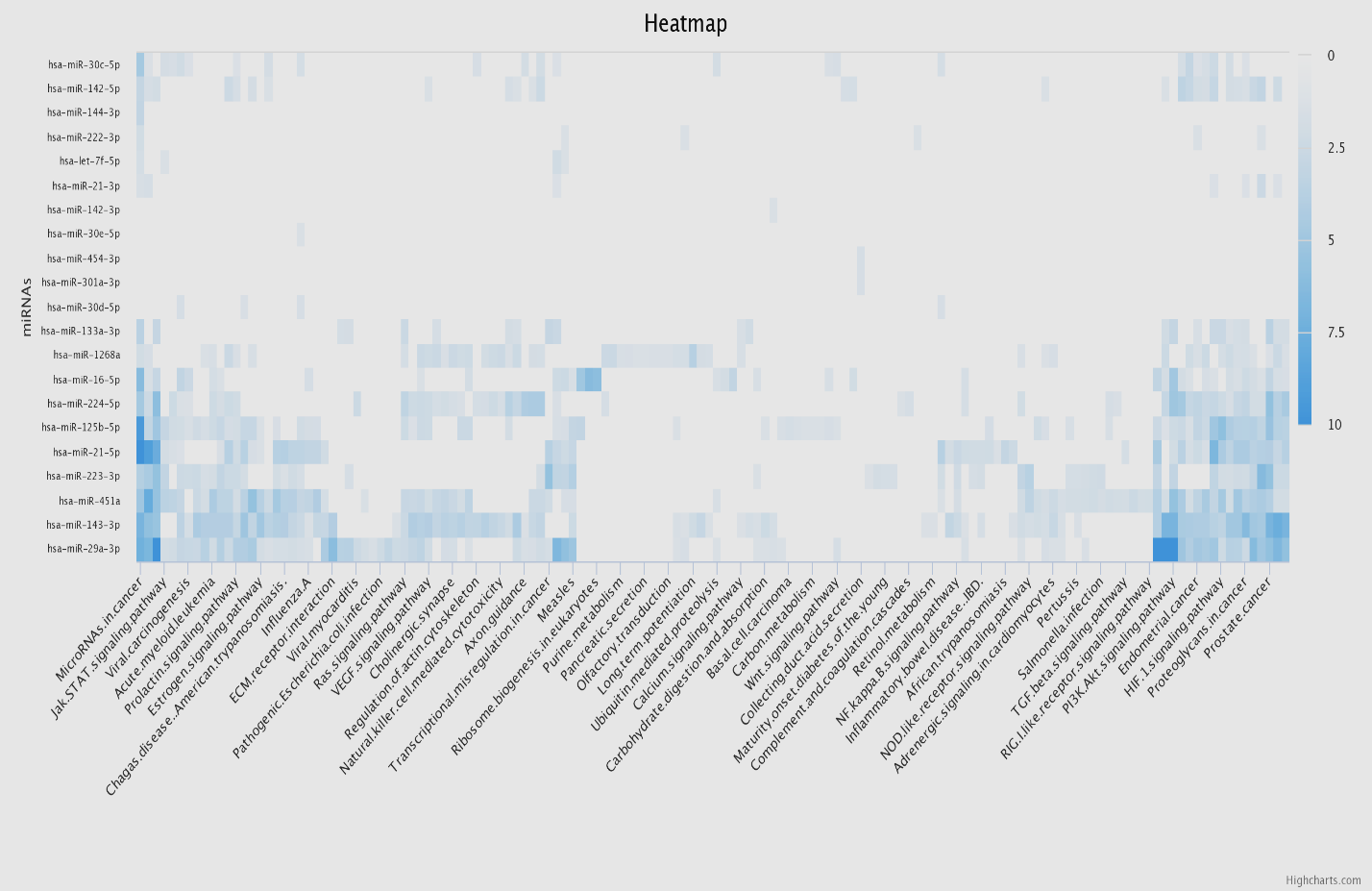


**Supplementary Figure S8. NF-kB expression in Schwann cells (SCs) upon lipopolysaccharide (LPS) stimulation. (**A) Experimental design of SCs exposed to different concentrations of LPS (100, 500, and 1000 ng/mL) for time-course study at 2, 8, 16, and 24 hours of exposure. (B) Representative fluorescent images of SCs at each time point, identifying localization of NF-kB with nucleus. Scale bar: 50 µm. (C) Hoescht staining identifies nucleus. Scale bar: 100 µm. MATLAB program identifies perimeter of each cell and localization of NF-kB within each cell with DAPI mask. Scale bar: 100 µm. (D) Comparison of relative NF-kB localization quantified utilizing MATLAB program. C: control, 100, 500, and 1000 indicate LPS concentration. The numbers 2, 8, 16, and 24 indicate the time by hours. For example, 2-C, 2-100, 2-500, and 2-1000 are for 2-hour time point. N=100-200, *, **, and *** indicate p<0.05.

**
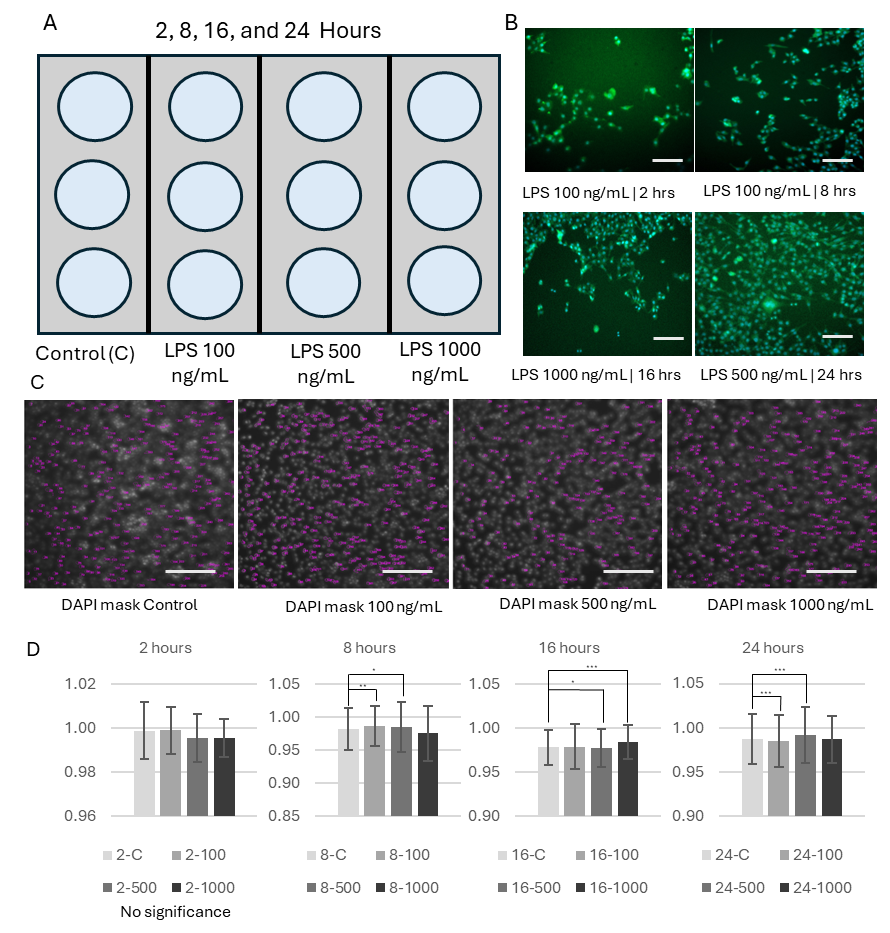
**
